# Mendel’s pea crosses: varieties, traits and statistics

**DOI:** 10.1186/s41065-019-0111-y

**Published:** 2019-10-31

**Authors:** T. H. Noel Ellis, Julie M. I. Hofer, Martin T. Swain, Peter J. van Dijk

**Affiliations:** 1 School of Biological Sciences, University of Auckland, Auckland, New Zealand; 2 Present address: Department of Metabolic Biology, John Innes Centre, Norwich, UK; 3 IBERS, Aberystwyth University, Aberystwyth, UK; 4 Keygene N.V, 6708 PW Wageningen, The Netherlands

**Keywords:** Gregor Mendel, Pea varieties, RA Fisher, Statistical controversy

## Abstract

A controversy arose over Mendel’s pea crossing experiments after the statistician R.A. Fisher proposed how these may have been performed and criticised Mendel’s interpretation of his data. Here we re-examine Mendel’s experiments and investigate Fisher’s statistical criticisms of bias. We describe pea varieties available in Mendel’s time and show that these could readily provide all the material Mendel needed for his experiments; the characters he chose to follow were clearly described in catalogues at the time. The combination of character states available in these varieties, together with Eichling’s report of crosses Mendel performed, suggest that two of his F3 progeny test experiments may have involved the same F2 population, and therefore that these data should not be treated as independent variables in statistical analysis of Mendel’s data. A comprehensive re-examination of Mendel’s segregation ratios does not support previous suggestions that they differ remarkably from expectation. The χ^2^ values for his segregation ratios sum to a value close to the expectation and there is no deficiency of extreme segregation ratios. Overall the χ values for Mendel’s segregation ratios deviate slightly from the standard normal distribution; this is probably because of the variance associated with phenotypic rather than genotypic ratios and because Mendel excluded some data sets with small numbers of progeny, where he noted the ratios “deviate not insignificantly” from expectation.

## Introduction

Mendel’s genetical studies have received a considerable amount of attention since the 150th anniversary of his 1865 lectures and 1866 paper [1, 2, 3, 4, 5, 6]. This attention broadly sought to understand Mendel’s work and intellectual position, but did not directly discuss his experimental plans nor seek to resolve the controversy, initiated by Fisher [7], concerning the statistical analysis of Mendel’s data. Fisher’s criticism had been reviewed several years earlier, in the book ‘Ending the Mendel-Fisher Controversy’ [8]. The central issue of the Mendel-Fisher controversy is Fisher’s claim, repeated by later authors, that Mendel’s results were closer to his theoretical predictions than should be expected. This supposed anomaly was especially noticeable in Mendel’s analysis of F3 families because Fisher thought Mendel had made a mistake in predicting these segregation ratios. Although Franklin et al. [8] concluded their book on a favourable note, some doubt about Mendel’s results remained, notably in Edwards [9] which reassessed Fisher’s earlier analyses (reproduced from [10]), and essentially upheld Fisher’s criticism.

Fisher’s [7] paper discussed the general plan of Mendel’s experiments, undertook a statistical analysis of Mendel’s data and asked whether Mendel should be taken literally. In Fisher’s “attempted reconstruction” of Mendel’s experiments, the number of plants grown in each year is estimated, assuming that the experiments were conducted in the order they were presented in Mendel’s 1866 paper [11], [3]. On completion of his statistical analysis, Fisher proposed:

"Although no explanation can be expected to be satisfactory, it remains a possibility among others that Mendel was deceived by some assistant who knew too well what was expected. This possibility is supported by independent evidence that the data of most, if not all, of the experiments have been falsified so as to agree closely with Mendel's expectations."

Although Weldon [12] was the first to suggest that Mendel’s data “accord so remarkably with Mendel’s summary”, Fisher is usually taken as the source of doubt concerning Mendel’s scientific credentials. Here, we describe several aspects of this controversy, noting particularly the connection between the reconstruction of Mendel’s experiments and the statistical analysis. Varieties, linkage and statistics have also been discussed by Fairbanks and Rytting [13] – (reproduced in [8]) and several authors ([9, 14, 15, 16, 17, 18, 19, 20], and citations therein) have attempted to propose biological or methodological reasons why Mendel’s results attracted the attention of Weldon and Fisher. Here we add several new sources of information and additional statistical treatments of the data; we note that Fisher facilitated his statistical analysis by constraining the possible approaches that Mendel might have taken, but we argue that Fisher’s model is, unjustifiably, too constrained.

### Plant materials available to Mendel

The link between Fisher’s reconstruction and his statistical analysis was essential for his interpretation but, as with any scientific paper, it should not be assumed that the chronology of Mendel’s experimentation is reflected in the sequence in which the experiments are presented.

Another important issue for statistical analysis is the number of characters which segregate in a given cross, because this relates to the independence of segregation ratios. At the beginning of section 8 of the 1866 paper, where Mendel began his discussion of the joint segregation of more than one character, he wrote:

“In the experiments just reviewed, plants were used that were different in one essential [wesentlichen] trait only” p18^1^

This has usually been taken to mean that Mendel’s crosses were made between plants that differed in only one of the seven traits that he investigated, but these plants could differ for other traits not under examination, for example flowering time or seed size. Fisher [7] doubted that Mendel had considered only one trait at a time, a doubt that had been raised before by Bateson [21], and discussed later by Corcos and Monaghan [22] and Di Trochio [23]. A relevant issue therefore, is what material was available to Mendel for his experiments.

Most of Mendel’s varieties originated from abroad [5]; according to an article in the local newspaper ‘Tagesbote’ [24], gardeners in Brünn acquired vegetable seeds from German seed dealers. From Eichling [25] and from a later seed-order we know that Mendel was in contact with Benary in Erfurt, Germany [26]. Forty seven pea varieties available at the time in Germany were cultivated, described, and classified by Bouché [27]. A subset of 15 of these, where the status of several Mendelian characters can be determined, is described in Additional file 1: Table S1.1. Bouché [27] used 4 of Mendel’s 7 traits to classify all the varieties (seed shape, plant length (=height), pod type and flower colour), and in addition he described the colour of the seeds, pod colour (green vs yellow) and flower position (terminal vs axial) for a few of the varieties, completing the set of seven character states that Mendel used. Mendel’s use of contrasting pairs of character states, which distinguishes him from other hybridizers, may have been a consequence of the way that pea variety lists, such as Bouché’s and others [28, 29–32], were organised.

Among the 15 varieties for which we could obtain the most information, there are 105 different parental combinations that could be used in crossing. Of these, 21 would segregate for a single Mendelian character. For each of Mendel’s characters, at least one cross can be found in which the character alone segregates (Additional file 1: Table S1.2). Crosses for which 2, 3, 4 or 5 characters would segregate in the F2 can be found, and for three crosses, if it is assumed that pod colour is green where this is not specified, 6 characters would segregate. 70 of these crosses would segregate for either seed shape (*R* vs *r*) or cotyledon colour (*I* vs *i*) and 16 would segregate for both, and so could have been used directly of Mendel’s bifactorial crosses involving these characters. Of these 16, 7 would also segregate for flower colour (*A* vs *a*) as used in Mendel’s trifactorial cross. So, contrary to Di Trocchio [23] or Corcos and Monaghan [22], this subset of varieties, available in Germany in Mendel’s time, could have been used directly for all of Mendel’s pea crosses.

Mendel had a breeding program on peas, beans and cucurbits intended to produce new varieties with improved characteristics [5] and if the varieties Bouché [27] described are typical, then $$ \raisebox{1ex}{$2$}\!\left/ \!\raisebox{-1ex}{$3$}\right. $$ of the breeding crosses would have segregated for seed shape and/or cotyledon colour. With his background in meteorology and physics and his appreciation for numbers, Mendel may have noticed that green or wrinkled seeds were typically ca. ¼ of the F2 in these crosses. Where both segregate a ca. ¼ of both the green and yellow seeds would be wrinkled and ca. ¼ or both the round and wrinkled seeds would be green. As Mendel had studied combinatorial theory under Ettinghausen in Vienna [33] this may have piqued his interest.

Fisher’s [7] reconstruction of Mendel’s experiments assumed that the initial crosses were monofactorial and the bi- and tri-factorial crosses were undertaken later. However, this was not necessarily the case because, even among 15 of Bouché’s varieties, the material for any of Mendel’s crosses would have been available throughout his 8 years of experimentation. The 2 years of trialling that Mendel undertook with 34 varieties [11, 3] would have provided information about purity of seed lots, similarity of varieties, and the stability (both phenotypic and genotypic) of the character states of these varieties. This would undoubtedly have assisted in his identifying the 22 varieties that he chose to cross for his experiments and the (7) characters he chose to follow in their offspring.

Whether any of the experiments, other than the bi- and tri-factorial crosses, involved more than one of Mendel’s characters, is relevant to the F2:F3 experiments (where the genotype of F2 individuals of the dominant class was inferred from the behaviour of their selfed progeny in the F3). This is because monofactorial ratios may be obtained by combining information from multiple crosses where additional characters segregate, as was Bateson’s interpretation [21] of Mendel’s use of the word “essential” [“wesentlichen”, see above]. If this was, even in part, the way Mendel conducted his experiments then the χ and χ^2^ values should not be considered independent and should not be summed. Furthermore the two traits Mendel described as “length of the axis” and “shape of the pod”, are especially interesting because they may be controlled by linked genes.

It is thought that the “length of the axis” (tall vs dwarf) corresponds to the gene *le* [34, 35, 36]. The “shape of the pod” character corresponds either to *v* or *p,* where the recessive homozygote, for either gene, lacks a hard-walled cell layer such that the pods are soft and pleasant to eat. These are ‘sugar-pod’ or ‘mangetout’ types that had been grown for at least three hundred years before Mendel’s time [37]. The genes *le* and *v* are genetically linked, but *le* and *p* lie on different linkage groups (see [23, 38]).

From the list of 15 of Bouché’s varieties in Additional file 1: Table S1.1 it appears that, in Mendel’s time, the mangetout type (*vv* or *pp*) was available in either tall (*LeLe*) or dwarf (*lele*) varieties. In Mendel’s second letter to Nägeli (written in 1867, [39]) he wrote that he obtained a useful F2 plant type in 1859 from a cross he performed (presumably in 1857) between parents that differed in four characters. He also mentioned that this segregant bred true in the following generation (presumably 1860) and that it became a popular variety cultivated in the monastery [25, 5]. According to Eichling, Mendel said he obtained this tall “shelling type” (*LeLe, PP* & *VV*) from a cross between a “tall sugar-pod type” (*LeLe*, *vv* or *pp*) and a “bush”, “shelling type” (*lele, VV* & *PP*). This cross was followed through several generations and the F3 data would have been available in the autumn of 1860, which is slightly earlier than Fisher’s [7] estimation of the timing of Mendel’s first F2:F3 progeny test.

If the cross for the F2:F3 progeny tests for tall/dwarf and “shape of the ripe pod” and the four factor cross were the same, then the same F2 population would have been used to score two characters in the F3 progeny tests. Furthermore, if this involved *le* and *v* (rather than *p*) then the linkage between *le* and *v* (recombination fraction ca. 11%, [40]) means that their segregation would not have been independent: in the F2 generation, *Lele* heterozygotes would often (ca. 81%) also be *Vv* heterozygotes.

**Fig. 1:**
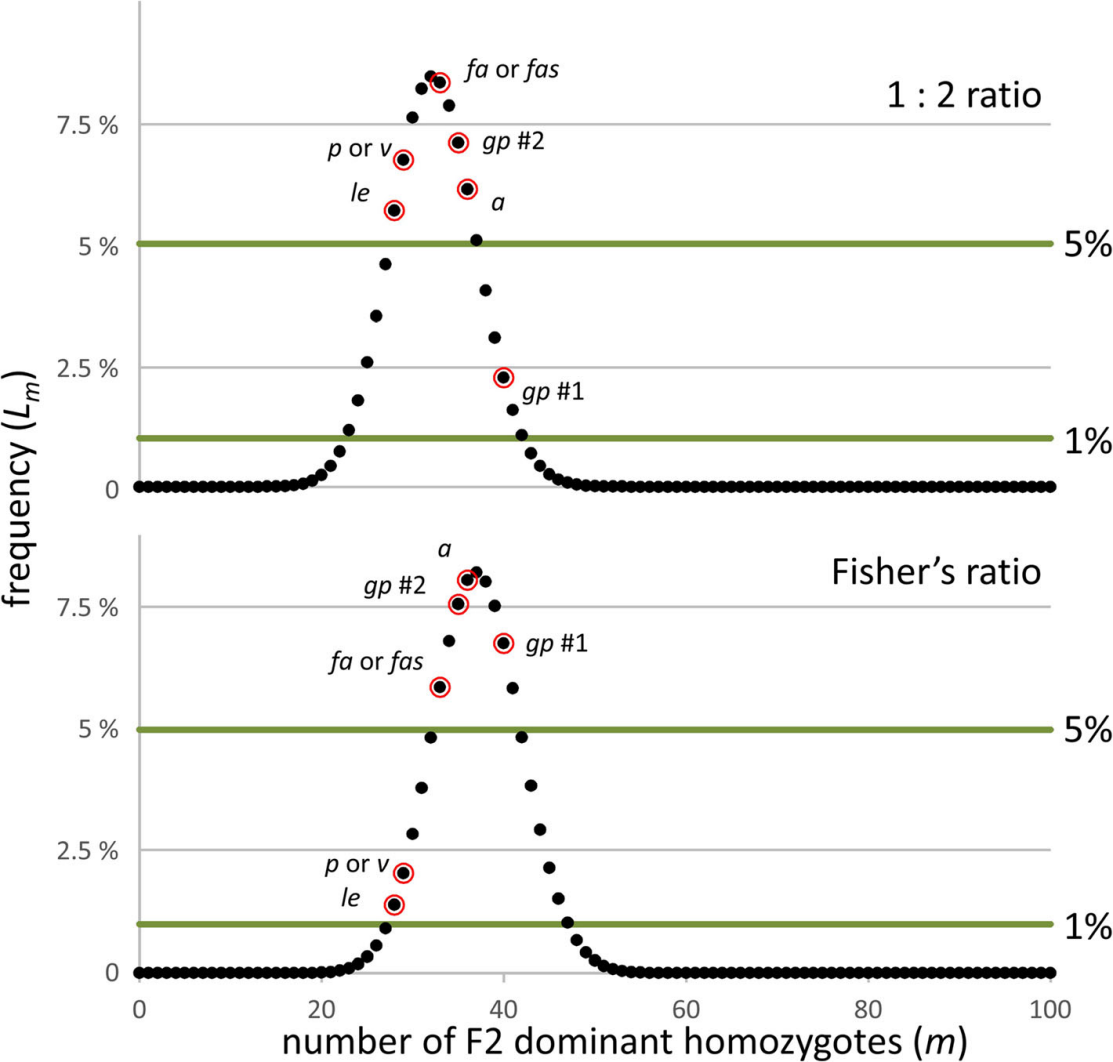
Frequency distribution of segregation ratios. The number of dominant homozygotes (*AA*) in the F2, *m* (x axis), among 100 F2 individuals of the dominant class (*AA* and *Aa*) (Additional file 1: Table S1.3) is plotted against the frequency with which this number is expected to occur. This frequency corresponds to the likelihood, *L*_*m*_*,* (y-axis) with which that number occurs as determined by the binomial distribution; these values were obtained in Excel using the function BINOM.DIST. The values that Mendel obtained are ringed and the gene involved is indicated, see Ellis et al. [13] for an explanation of the gene symbols. The upper panel is the frequency expected given a 1: 2 ratio, and the lower panel is the frequency distribution based on Fisher’s ratio

In the four factor cross discussed by Eichling [25] the linkage was in repulsion, so the occurrence of double homozygous recessive types in the F2 would be rare (see Additional file 2, p5). If this was in fact what happened, Mendel would have had difficulty in noticing that they behaved differently from the others he studied, i.e. as unlinked characters. If such an F2 population was used to generate an F3 in which both *le* and *v* were scored, then many (but not all) of the heterozygotes identified for each character would be the same F2 individual, consistent with them having similar segregation ratios as seen in Fig. 1. In this case the χ^2^ values should not be considered to be independent as is necessary for Fisher’s analysis and interpretation.

### Mendel’s F2:F3 experiments

#### Datasets and analyses

Here we will discuss the segregation ratios presented in Mendel’s 1866 paper [11] using both χ and χ^2^ statistics, much as Edwards [9, 10] and Weir [41] did, but with some differences as discussed. Edwards helpfully partitioned Mendel’s data into a set of single factor segregation ratios, and presented a table of the corresponding χ values, noting that the χ values were expected to have a mean of 0 and variance of 1, while the χ^2^ values had an expected mean of 1 and variance of 2. This approach made Mendel’s data as a whole easy to examine. A modified version of Edwards’ table of segregation ratios is given in Additional file 1: Table S1.3 and the details of the statistical analyses are presented in Additional file 2 which also considers the estimation of the actual allele frequencies in Mendel’s experiments; by chance alone the *A* and *a* alleles are not necessarily exactly equally abundant.

For the differentiation between *AA* and *Aa* genotypes in the F2, it is necessary to examine the F3 progeny of each individual plant; on average 1 in 4 of F3 progeny of *Aa* genotypes will be *aa* homozygous recessive but none occur in the selfed progeny of *AA* genotypes*.* If there are very few F3 seed it is possible that, by chance alone, no recessive types will appear in the F3, so for small F3 families some *Aa* genotypes may therefore be misclassified as *AA*. This is the core of Fisher’s main criticism of Mendel’s data.

#### Seed characters and Mendel’s first set of F2:F3 progeny tests

Analysis of the segregation of seed characters (*R* vs *r* and *I* vs *i*) is relatively easily done, because the F3 seed occurs in the pods on the F2 plants. In these two experiments Mendel was able to examine all his F2 plants and all their F3 seeds; his total of 1084 F2 plants likely involved the classification of about 30,000 F3 seeds. In his first two experiments, Mendel provided 24 examples of single plant data, for which the seed yield (number per plant = 44.21 ± 21.74, μ ± SD) was slightly higher than for the two experiments as a whole. This mean and standard deviation suggests that among the 1084 F2 plants for the F3 progeny test, about 60 plants would have had 10 or fewer F3 seeds. Therefore we can estimate that among these 1084 plants the frequency of misclassifying *Aa* genotypes as *AA* is low (ca. 5%). In this situation it is reasonable to consider that the genotypic segregation ratio should approximate to the expected 1: 2 ratio (*AA*: *Aa*).

#### Plant characters in Mendel’s first set of F2:F3 progeny tests

**Fig. 2:**
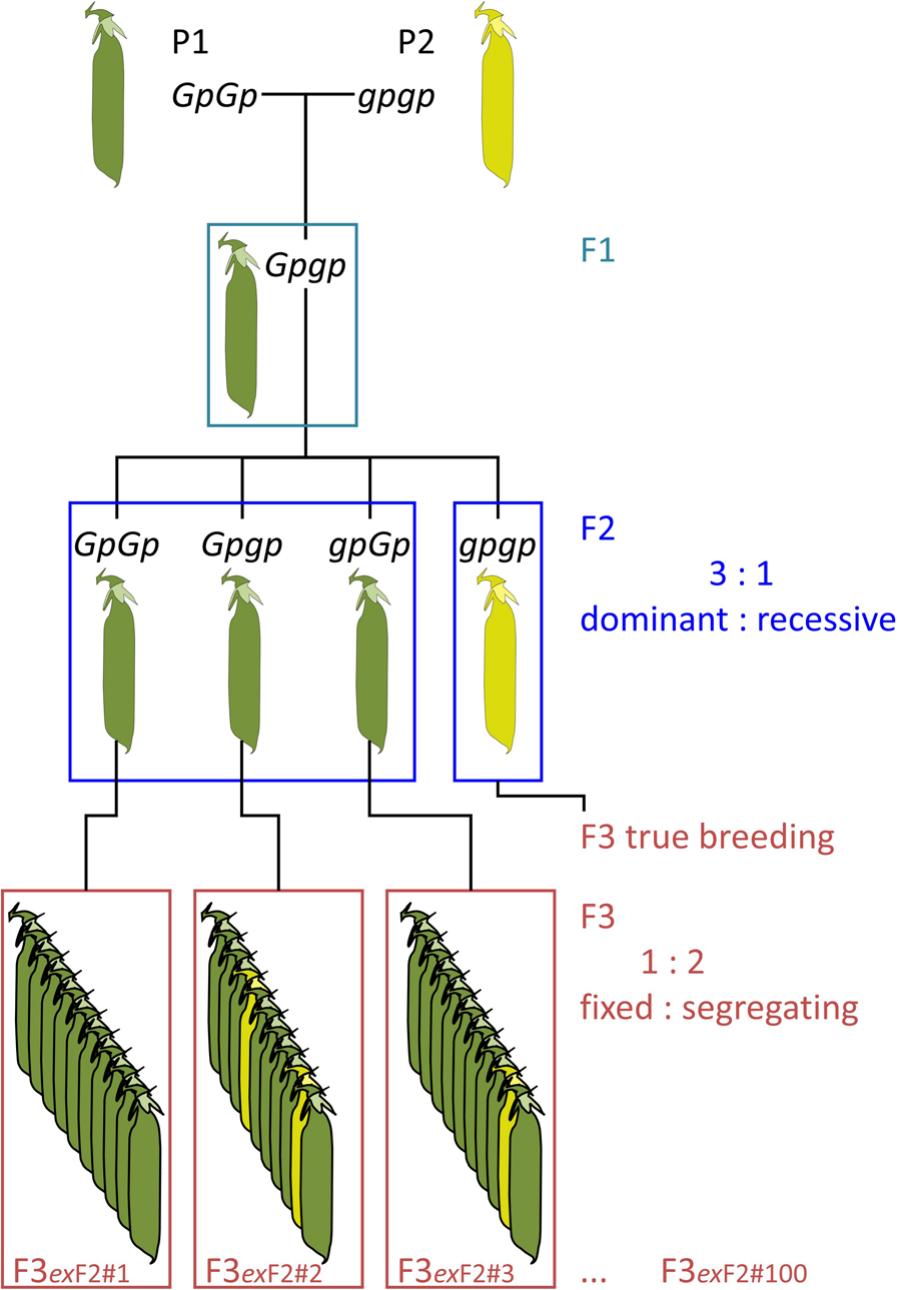
Experimental design. This figure represents Mendel’s F2:F3 experiment to determine the genotype of individuals of the dominant (green) class in the F2 by examining the segregation of green vs yellow pods (*Gp* vs *gp*) in their F3 selfed progeny. The gene symbols are as described in Ellis et al. [13]

The seed characters discussed above can be scored using the F2 seeds that are in the pods of the F1 plant. In order to score other characters (plant height, pod colour etc.) it is necessary to germinate these seeds and grow the plant until the relevant vegetative or reproductive structures are produced. Here we follow Edwards [10] designation of these as ‘plant characters’. It is important to realise that seed coat (testa) colour is one of these ‘plant characters’ because the testa is maternal tissue. Mendel limited the number of F3 plants that he needed to grow in this experiment in two ways: first, he limited the study to the selfed progeny of 100 F2 individuals of the dominant class, and secondly, for each F2 individual, “10 plants were cultivated” (Fig. 2). Fisher reasoned that the expected ratio of *AA*: *Aa* is 1: 2 and in the F3 ¼ of the offspring of a heterozygote should be *aa*. This follows Mendel’s description, but Fisher noted that the chance that 10 F3 segregants, from a selfed heterozygote, do not have any recessive segregants is (1 - ¼)^10^. Thus, because of misclassification, Fisher expected the *AA*: *Aa* ratio should be 1: 1.6964 (Fisher’s ratio) rather than a 1: 2 ratio. The way this ratio is obtained and applied to Mendel’s data is discussed in Additional file 2 and his experimental design is discussed in more detail in Additional file 3.

Fisher’s estimate of the segregation ratio as a consequence of misclassification is dependent on the assumption that exactly 10 seeds of exactly 100 plants were sown and characterised, and this is extremely unlikely considering the germination rates that can be deduced from Mendel’s data. Mendel gave two examples of the survival or germination rates, and these suggest that his success rate in producing plants from seeds was 98% or perhaps a little less (Additional file 3) so if he planted 10 seeds the chance that they all survived is *s* = 1–0.98^10^; the chance that all 10 seeds survive in all of 100 plantings is (1 - *s*)^100^ ≈ 10^− 9^ so it seems unlikely that exactly 10 seeds of exactly 100 F2 individuals were planted and grown. Fisher’s criticism of these F2:F3 experiments is that Mendel’s results are closer to the 1: 2 ratio than to Fisher’s 1: 1.6964 ratio. As Franklin [42] wrote in his summary of the “Mendel-Fisher Controversy”:

“Fisher commented, 'It is interesting that Mendel’s original results all fall within the limits of probable error; if his experiments were repeated the odds against getting such good results is about 16 to one. It may just have been luck; or it may be that the worthy German [sic] abbot, in his ignorance of probable error, unconsciously placed doubtful plants on the side which favoured his hypothesis' (qtd. in Norton and Pearson 1976, 160). Fisher later changed his mind and attributed these results to the work of an assistant.”

It should be noted that Mendel could not possibly obtain a result that was exactly either of these ratios because the ratio he obtained must consist of two integers that sum to 100.

Given this constraint, we can derive the expected frequency of each possible ratio and see how the distribution of segregation ratios Mendel obtained compares to their expected frequencies. We can define the probability of an individual F2 being classified as a homozygous dominant as *p*, where *p* is either $$ \raisebox{1ex}{$1$}\!\left/ \!\raisebox{-1ex}{$3$}\right. $$ or is given by Fisher’s relationship: *p* = ($$ \raisebox{1ex}{$1$}\!\left/ \!\raisebox{-1ex}{$3$}\right. $$) + (¾)^10^($$ \raisebox{1ex}{$2$}\!\left/ \!\raisebox{-1ex}{$3$}\right. $$). It follows therefore that the likelihood, *L*_*m*_, of declaring exactly *m* homozygous dominant individuals among 100 F2 s is given by the binomial formula:

1 $$ {L}_m=\left(\frac{100!}{m!\left(100-m\right)!}\right){p}^m{\left(1-p\right)}^{100-m} $$

This relationship is plotted in Fig. 1 and Mendel’s individual results are indicated as circled points. From this binomial distribution it is clear that two values, for *le* and *v* (or *p*), are significantly different from Fisher’s expectation at the 5% level, but not the 1% level. According to the 1: 2 genotypic ratio, Mendel’s first test of the pod colour character (*gp*) is significantly different from expectation at the 5% but not 1% level. For this binomial distribution, the most likely ratio occurs in only about 8% of cases. According to Fisher’s ratio, the least likely of Mendel’s values (for *le*) is at *L*_*m*_ ≈ 0.0140, but taking account of Mendel having performed six experiments, at least one such value among six occurs with a frequency of 1 - (1 - *L*_*m*_)^6^ ≈ 0.0811 (or about 8%; for the combined data of all six experiments Fisher estimated finding ratios with this deviation from expectation once in 16 trials). Given the multiple tests, none of Mendel’s observed segregation ratios is significant, even at the 5% level.

We should note that the segregation ratios for *le* and *v* (or *p*) are very similar as would be expected if they derived from the same heterozygotes in the same seed lot. In fact these two ratios are more alike than are the repeat trials with pod colour (*gp*), consistent with their being the same actual plants rather than different samples from the same F2 (see above).

Mendel provided F2 data for these experiments, from which, assuming random mating of the gametes, the allelic ratios in these particular seed batches can be estimated, and this allows a comparison between the F2 and estimated F3 ratios (Additional file 2). From these data we can conclude that the F2 and F3 ratios are within the expected range of one another, but that for *le* and *v* (or *p*) the deviation is greatest, and again these two characters behave in a very similar way, consistent with the underlying genes being linked. This suggests that Mendel’s gene was *v* rather than *p.*

#### The F2:F3 analysis in Mendel’s trifactorial experiment

In the trifactorial experiment, described in section 8 of the 1866 paper, Mendel did not state how the F2 genotypes were determined for seed coat colour (*a*, [43]); the approach taken is simply stated as “further investigations”. As in the bifactorial cross, Mendel would have had an abundance of F3 seed on his mature F2 plants for the determination of seed characters, and Edwards [9, 10] appropriately gives an expectation of a 1: 2 ratio for these characters. Although ‘seed coat colour’ sounds like a seed character, the seed coat (testa) is maternal tissue; the F3 seeds must be cultivated as plants in order to determine whether the F2 was *AA* or *Aa*. Mendel did not state how many F3 plants were cultivated in this experiment; it could be that he used 10 F3 plants, as in the experiments discussed in the previous section, but he could have grown fewer or more. We can note Mendel’s comment “Among all the experiments, this one asked for the most time and effort”, suggesting that this was a large experiment.

Mendel knew that he did not have to wait until the seeds were set to determine the seed-coat colour in the F3 progeny; the character could be scored at the seedling stage according to the pigmentation of the leaf axil (“axilla”), or at the flowering stage according to flower colour.

" ... the colour of the standard appears violet, that of the wings purple, and that of the pedicels at the leaf axils marked red. " p8

The convenience of using leaf axil colour would have been obvious, so Mendel could have examined many more than 10 F3 segregants without needing to cultivate all the plants to maturity.^2^ In this trifactorial experiment it is therefore possible that a 1: 2 ratio might better represent the expectation than Fisher’s ratio, given that Fisher’s ratio is correct only for exactly 10 F3 plants. Edwards [9, 10] followed Fisher [7] in assuming that exactly 10 F3 plants were examined, so his tabulation of χ values is potentially incorrect.

The genes involved in the trifactorial experiment were *R* vs *r*, *I* vs *i* and *A* vs *a*, and Mendel reported the segregation of *A* vs *a* within each of the nine seed character classes (Table 1, Additional file 2: Table S2). The expected frequencies of these ratios, assuming Fisher’s 1: 1.6964 ratio or the 1: 2 ratio, are also given in Table 1, none of which is significant at the 1% level. Four classes have more *Aa* plants than a 1:2 ratio predicts; the abundance of the *aa* class in the F2 of this batch of seed suggests the frequency of the *a* allele is 0.51 and therefore an excess of *Aa* plants is expected. Fisher’s ratio is based on the *a* allele frequency being exactly 0.5.

**Table 1:**
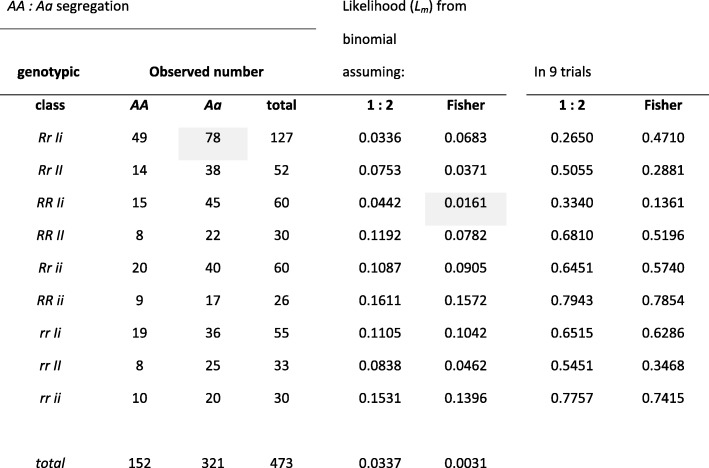
Anthocyanin pigmentation segregation in Mendel’s trifactorial experiment

The likelihoods of getting exactly the ratio *AA*: *Aa* as reported by Mendel were calculated as described by eq. 1 except that the value 100 is replaced by the number of segregants, given in the column “total”, and using the Excel function BINOM.DIST. The likelihoods (*L*_*m*_) are listed in the columns “Likelihood from binomial assuming:” in which the two columns correspond to different expectations; the 1: 2 ratio, and Fisher’s ratio. The columns under “In 9 trials” give the corresponding likelihoods in a series of nine trials, there being nine categories in these data, this is estimated as [1 - (1 - *L*_*m*_)^9^]

The shaded values (i) are the case in the most abundant class (*Rr Ii*, 127 plants) where there are fewer *Aa* plants than Fisher’s ratio predicts (78 vs 84.67), and (ii) the lowest likelihood of the nine *AA*: *Aa* segregation ratios (1.6% for the *RR Ii* class of 60 plants)

There are two points to consider about these ratios. The first is whether or not to assume that Mendel used exactly 10 F3 individuals selfed from each F2 and the second is whether to assume that the *A* and *a* alleles had an exactly equal ratio, or whether to estimate their ratio from the frequency of the *aa* class in the F2. Given these possibilities there are four ways to estimate the χ or χ^2^ values, and these are given in Additional file 2: Tables S2.4 and S2.5). If we consider the distribution of χ values, the results most closely fit the expectations (mean of χ = − 0.1679 vs expectation of 0) when the allele frequency is based on the observed frequency of the recessive class in the F2 seed lot and when it is assumed that misclassification did not arise from scoring exactly 10 F3 individual selfed progeny of each F2 plant. The simplest interpretation is that this reflects the experiment which Mendel performed. However, if the χ^2^ values are considered, the best fit is when the allele frequency is based on the observed frequency of the recessive class in the F2 seed lot and assuming the F2 gamete ratio with misclassification (mean of χ^2^ = 1.0302 vs expectation of 1) from scoring exactly 10 F3 individuals (Additional file 2: Table S2.5). Either way the evidence is against the interpretation that the results were adjusted to fit to the 1: 2 ratio because, in this particular seed lot, they are closer to the estimate of the actual ratio rather than the theoretical 1: 2 ratio.

### Mendel’s segregation ratios in general

Fisher [7] additionally claimed that Mendel’s results, in general, are too close a fit to his expectations. Edwards [9, 10] conclusion upheld Fisher’s criticism and commented that “throughout the rest of his [Mendel’s] results there is a persistent lack of extreme segregations”. This comment is based on the distribution of 69 χ values, ranging from − 1.4237 to + 1.5811, and excluding those derived from the F3 progeny tests. Furthermore, the sum of the corresponding χ^2^ values is expected to be 69, but in Edwards [9, 10] the sum is 30.8138 (see also Additional file 1: Table S1.3 and Additional file 4). He commented that this was “highly remarkable on any interpretation of tests of significance”. Later Edwards [45] commented on the χ^2^ values from his complete analysis of 84 segregation ratios:

“The plain fact is that the expectation of χ^2^ is, for a binomial random variable, exactly 1, so 84 of them have an expectation of 84; if, then, 84 of them can only muster a total of 48.91, something is awry. One can applaud the lucky gambler; but when he is lucky again to-morrow, and the next day, and the following day, one is entitled to become a little suspicious.”

In this analysis Edwards [9, 10] did not use data from one of Mendel’s paragraphs that has a bearing on extremes of segregation ratios:

“As extremes in the distribution of both seed traits on one plant, there were observed in the first experiment 43 round and only 2 angular seeds on the one hand, and 14 round and 15 angular ones on the other. In the 2nd experiment 32 yellow and only 1 green seed, but also 20 yellow and 19 green ones.” p13

For each of the first two experiments Mendel gave the number of seeds with dominant and recessive phenotypes for (i) the experiment as a whole, (ii) for the first ten plants (as an example of variation) and (iii) for two extremes of segregation, one where the number of dominant and recessive seeds was nearly equal and another where the number of recessive seeds was very low. In so doing Mendel provided a general picture of segregation ratios, some typical examples of variation, and an illustration of how extreme this variation might be.

Inclusion of these extreme segregation ratios, given by Mendel, adds four segregation ratios to Edwards’ table and also changes the numerical value of the first two entries. The aggregation of these extremes and the “ten examples” that Mendel gave (Additional file 1: Table S1.3) corresponds to a data set of 88 segregation ratios where the χ^2^ sums to 90.6 or 86.2 depending on the way the trifactorial experiment is treated (Additional file 1: Table S1.4d and S1.4f) these are clearly much closer to the expected sum of 88 than Edwards’ [9] value of 48.9.

**Fig. 3:**
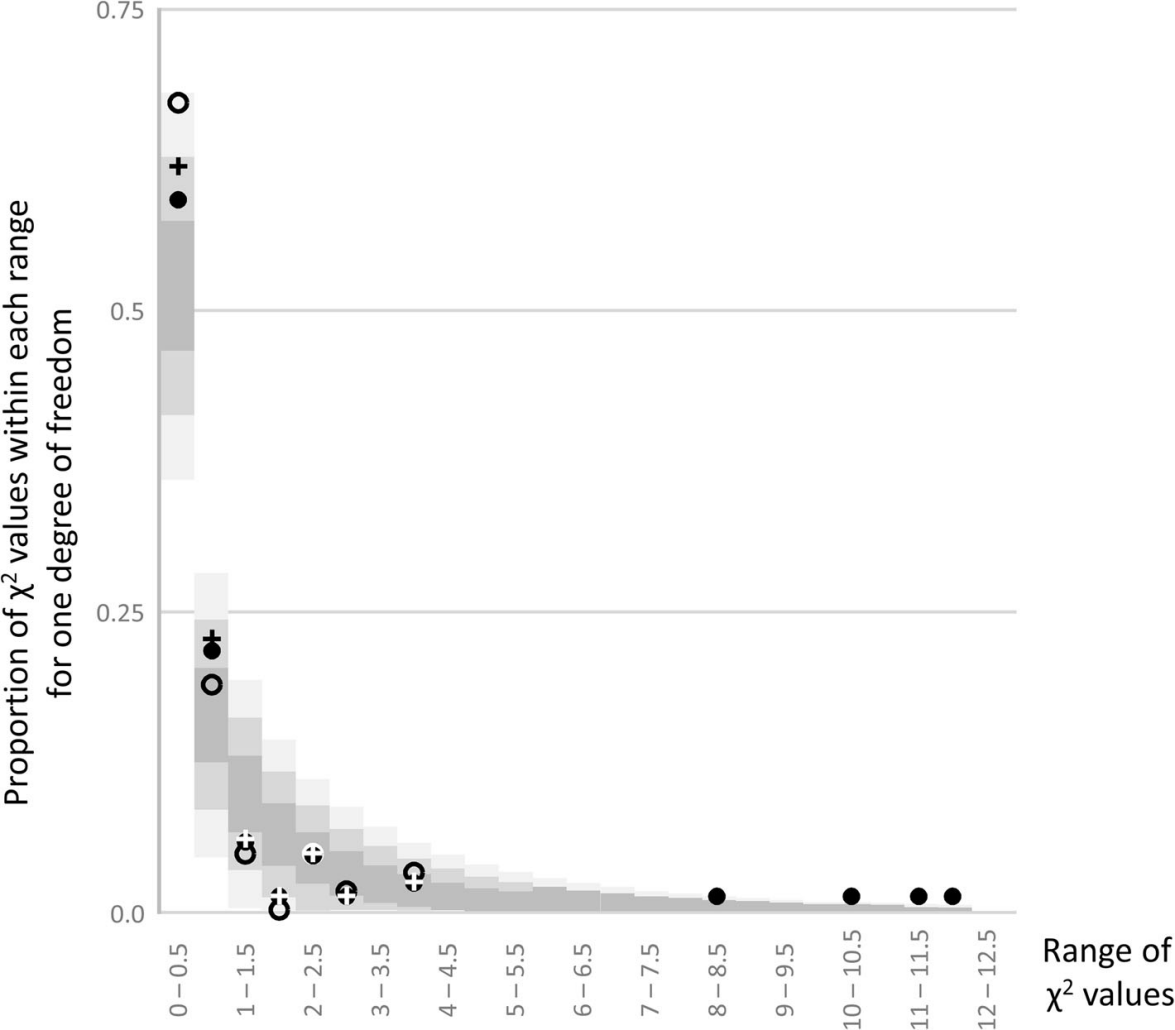
The frequency distribution of χ^2^ values. The frequency distribution of the proportion of χ^2^ values in a given range corresponding to phenotypic ratios in Mendel’s experiments is shown. The ranges of χ^2^ values are on the x axis and the frequency with which χ^2^ values in each range occur is on the y axis. For comparison the expected frequency distribution of χ^2^ values is also shown as shaded background bars. These are centred at the expected frequency and the three shades of grey correspond to ±1 (dark), 2 (lighter) or 3 (lightest) standard errors. Standard errors for the expected frequencies (*p*) were estimated as √(*p*(1-*p*)/*N*) where *N* = 88. Three sets χ^2^ values corresponding to Mendel’s data are plotted: Those with an open circle correspond to 64 values where the data were as Edward’s grouping, except that the data for Mendel’s experiments 1 and 2 are for the combined values. The second, marked as filled circles, correspond to 88 values which disaggregate the values of Mendel’s experiments 1 and 2 into all of the examples he gave for individual plants. The third set, with marked +, correspond to 84 values where experiments 1 and 2 disaggregate only the first 10 plants of each experiment as in Edwards [8]. The data corresponding to these values are given in Additional file 1: Table S1.3. The expected frequencies were calculated in excel using the function CHISQ.DIST(*× 1*,*ν*,c)) - CHISQ.DIST(*× 2*, *ν*,c)) where *× 1* and *× 2* correspond to the range of χ^2^ values, *ν* = 1 (degree of freedom) and *c* = 1 (cumulative distribution)

In Edwards’ analysis, the seed numbers for the first ten plants were subtracted from the total so the eleven ratios that he examined per experiment did not include any double counting. If in addition to this, the “extreme” values are also extracted from the total and treated as two additional ratios, then there are 13 ratios to analyse in each of these two experiments. It is not surprising that the χ^2^ values for the “extreme” ratios are high, but removing them from the total also increases the χ^2^ value for the remainder (Additional file 1: Table S1.3 compare sets 2 and 3). It could be argued that using these four “extreme” examples in the χ and χ^2^ analyses is invalid because they are not a random selection of segregants. That argument necessarily also applies to the selection of 10 example plants in these experiments i.e. “The first ten members from both experimental series” and also because their average seed yield is greater than for the experiment as a whole. So instead of considering all 88 ratios, it is reasonable to examine the segregation ratios for the total number of seeds in these two experiments; in all, that is 64 ratios. The frequency distribution of these 64 χ^2^ values is shown in Fig. 3. For completeness, the actual observed, expected, χ and χ^2^ values for all of Mendel’s data, and relevant combinations of data, are given Additional file 1: Table S1.3. The distribution of χ^2^ values shows a deficiency of values in the middle of the range and an excess in the higher and lower values, but this is not statistically significant (Fig. 3).

An additional issue that has caused criticism [9, 10] of Mendel’s data is that the χ values derived from his results do not follow a standard normal distribution. This issue, together with the most appropriate grouping of Mendel’s data to examine, is discussed in Additional file 4. That analysis suggests that the χ values from the data presented in Mendel’s 1866 data [11] do not conform exactly to a standard normal distribution. This is consistent with his account, where he explained why he presented some data and not others, so it is not appropriate to assume the data was selected at random as would be required for it to fit the expected cumulative χ distribution in normal probability plots.

## Concluding remarks

Fisher’s [7] reconstruction of Mendel’s experiments is a helpful illustration of the scale of his experiments and the logistics of how they may have been conducted, but it makes the assumption that Mendel’s experimental schedule followed the sequence in which Mendel presented his experiments. We show that this is not necessarily the case as it is likely that, at the outset, Mendel had to hand varieties that segregated for any number and many combinations of the characters he studied, so the experiments could have been performed in any temporal order. Fisher [7] reports Bateson as having suggested that Mendel may have, for example, collected single factor segregation data from crosses in which more than one of his factors segregated. Fisher rejected Bateson’s suggestion because it would mean Mendel had taken “excessive and unnecessary liberties with the facts”. We present some evidence to support Bateson’s interpretation in the segregation of *le* and *v* in the F2:F3 experiments. Indeed, Mendel’s use of the word “wesentlichen” (“essential” p18) can be taken to support Bateson’s notion more generally. If this genuinely describes Mendel’s experiments then the statistical analyses of his data should not consider the experiments as independent; invalidating a key assumption of Fisher’s analysis.

We have shown that several features of Mendel’s experimental data have been misinterpreted. First, there is nothing unexpected in Mendel’s observed F3 segregation ratios, from which the F2 genotypes were deduced. It is true that on average Mendel’s segregation ratios are closer to 1: 2 than Fisher’s 1: 1.6964 expectation, but they have to be the ratio of two numbers that sum to 100 (F2 plants) and Fisher’s ratio (Fig. 1) ignores the fact that Mendel’s F2 ratios were not from idealised populations, but from actual F2 populations where the segregation ratio was not exactly equal for the two alleles (Additional file 2). Based on the F2 ratios in Mendel’s actual seed lots, the ratios should not be Fisher’s ratio (Table 1, Additional file 2: Figure S2.1).

Secondly, Mendel’s data as a whole have been proposed to be deficient in extreme segregation ratios [9–10], but whether or not there is a deficiency of χ^2^ values in a particular range depends on the way the segregation ratios are combined or disaggregated. For Fig. 3 the data have been combined in what seems the most consistent way and in this analysis there is a deficiency of segregation ratios within the range 0.75 < χ^2^ < 2.25 (Fig. 3, Additional file 4: Table S4.1), which could alternatively be described as an excess of χ^2^ values below *and* above this range. Therefore, the proposed deficiency of χ^2^ values appears to be more an artefact of the way that later analyses aggregated the data, rather than a problem with the actual data.

A close examination of Mendel’s data shows that the χ values deviate slightly from a standard normal distribution, meaning that the results he presented are not a random selection from the idealised segregation of genotypic ratios. Mendel did not claim to have presented a random selection of his data. He explicitly stated that this was not the case when, in describing the first two experiments he wrote, “The first ten members from both experimental series [that] may serve as an illustration” (p13) and again in providing examples of contrasting “extremes” (p13) of segregation ratios. He also alluded to data that was not presented when he wrote:

“In addition, several experiments were carried out with a smaller quantity of experimental plants, in which the remaining traits were joined in hybrid fashion in twos and threes; all gave approximately identical results.” p22

From these comments it is clear that Mendel had data which he did not present, and indeed he explicitly stated that this was the case. So we know a priori that any analysis of the data he presented is incomplete and we also know that it is not a random selection from the data he collected. Mendel also stated that the experimental data he did not present was from experiments with relatively small numbers of individuals. Including these would have consumed a considerable amount of time in his oral presentation, and as Mendel wrote to Nägeli “The paper which was submitted to you [Nägeli] is the unchanged reprint of the draft of the lecture mentioned; thus the brevity of the exposition, as is essential in a public lecture.” [39]. Mendel further noted that among these there was considerable deviation from the expected ratio.

“With a relatively small number of experimental plants, the result could then be approximately right only and **deviate not insignificantly** in individual cases.” p 39 , our emphasis.

As Mendel made these two points openly and clearly, it is perhaps not surprising that the data he did present has segregation ratios closer to the median expectation of the χ^2^ than to its mean.

We should also realise that the mean and variance of the χ and χ^2^ values is for the genotypic ratios, but Mendel’s data are for phenotypic ratios from which the genotypes were inferred. We should expect some genotyping errors which would increase the variance of the expected χ distribution, perhaps contributing to the slight deviation of the χ values from the standard normal distribution in normal probability plots ([9–10], Additional file 4).

Statistical criticism of Mendel’s data has been a pernicious feature of the discussions of his work and has done great damage to the reputation of one of history’s most insightful biological scientists. Although “mud sticks” [46], a close inspection of the claims against Mendel reveals them to be false in their own terms. Statistical tests [9–10, 7] purportedly reveal Mendel’s results to be those of a persistently “lucky gambler”, but after a reconsideration of how to partition Mendel’s data for these analyses we show that there is nothing statistically remarkable about Mendel’s data. The “persistent lack of extreme segregation ratios” is not because Mendel failed to present them, it is because they have not been included in previous analyses. Mendel, on the other hand, consistently warned that the ratios he deduced were subject to fluctuation according to the laws of probability and gave ample warnings about the data he did not present.

Mendel’s 1866 paper [11] is exemplary, both in terms of its presentation and in its interpretation of numerical data; sadly an appreciation of the depth of its insights has been marred by a failure to accept its candour. Assuming the experiments to have been undertaken in an unrealistic manner solely to permit a specific statistical test [7], not surprisingly, shows that there is a slight deviation between the results and the statistical prediction. Rather than interpreting this as suggesting that the statistical analysis is in some way inappropriate (as is obvious from individual plant survival rates) the conclusion was drawn that Mendel’s experiments were a fraudulent attempt to prove a pre-existing hypothesis. It seems clear from this re-examination of Mendel’s data that the frequency distribution of genotypic classes is entirely as would be expected from his experiments and his comments on the data he presented in the presentations he made to the Natural Science Society of Brünn in 1865.

## Supplementary information

**Additional file 1:** Tabulated data. Variety descriptions and character segregation from [27] pea variety list and Mendel’s segregation ratios.

**Additional file 2: **The F2:F3 progeny tests for plant characters*.* Analysis of segregation ratios in Mendel’s F2:F3 progeny tests.

**Additional file 3:** Mendel’s F2:F3 progeny test experiments. Discussion of how Mendel’s F2:F3 progeny test experiments may have been performed given the material that would have been available.

**Additional file 4: **Normal probability plots*.* Analysis of the extent of deviation of segregation ratios in Mendel’s data [11] from a standard normal distribution.

## Data Availability

All data are presented in Additional file 1, except for the sets of standard normal variates used to estimate the confidence intervals in the figures of Additional file 4. These are available on request, or equivalent sets can be generated in R.

## Footnotes

1 For quotations from Mendel's 1866 paper [11], page numbers are given that refer to the BSHS translation [3], http://www.bshs.org.uk/bshs-translations/mendel/2016?page=#.

2 The ease with which this character can be scored depends on the allele at the *D* locus (see [34]), but Mendel drew attention to this property, so presumably the phenotype was clear to him. All of Mendel’s pea characters are illustrated by Margaret Stones in the book Legumes of the World Lewis et al. [44]
