# Supplementary material for: Mendel’s pea crosses: varieties, traits and statistics
Source: Hereditas. 2019 Oct 31;156:33. doi: 10.1186/s41065-019-0111-y (PMC6823958; doi:10.1186/s41065-019-0111-y)
Supplement: Supplementary file 1 — Additional file 1: Tabulated data. Variety descriptions and character segregation from [27] pea variety list and Mendel’s segregation ratios. [file 41065_2019_111_MOESM1_ESM.docx]

## Supplementary Table 1.1. Selected variety descriptions taken from Bouché (1854) in relation to Mendel's traits.

| **Variety** | **seed shape** | **cotyledon colour** | **flower colour** | **plant height** | **pod type** | **flower position** | **pod colour** |
| --- | --- | --- | --- | --- | --- | --- | --- |
| Holländische Schifferbse | Round *RR* Dominant | Yellow *II* Dominant | Purple-red *AA* Dominant | tall *Le* Dominant | Normal *PP* or *VV* Dominant | Axial *Fa* or *Fas* Dominant |  |
| Thurnston's Reliance | Round *RR* Dominant | Yellow *II* Dominant | white *aa* recessive | tall *Le* Dominant | Normal *PP* or *VV* Dominant | Axial *Fa* or *Fas* Dominant | Green *Gp* Dominant |
| Auvergne-Erbse | Round *RR* Dominant | Yellow *II* Dominant | white *aa* recessive | tall *Le* Dominant | Normal *PP* or *VV* Dominant | Axial *Fa* or *Fas* Dominant |  |
| Dolden-Erbse, Mummie-Erbse, Türkische-Erbse, Kronen-Erbse | Round *RR* Dominant | Yellow *II* Dominant | white *aa* recessive | tall *Le* Dominant | Normal *PP* or *VV* Dominant | terminal *fa* or f*as* recessive |  |
| Zuckerschote mit gelben Huelsen | Round *RR* Dominant | Yellow *II* Dominant | white *aa* recessive | tall *Le* Dominant | inflated *pp* or *vv* recessive | Axial *Fa* or *Fas* Dominant | yellow g*p* recessive |
| Weisse Schwert Zuckererbse | Round *RR* Dominant | Yellow *II* Dominant | white *aa* recessive | tall *Le* Dominant | inflated *pp* or *vv* recessive | Axial *Fa* or *Fas* Dominant |  |
| Bishop Longpod | Round *RR* Dominant | Yellow *II* Dominant | white *aa* recessive | dwarf *le* recessive | Normal *PP* or *VV* Dominant | Axial *Fa* or *Fas* Dominant |  |
| Waite's Queen of Dwarfs | Round *RR* Dominant | Yellow *II* Dominant | white *aa* recessive | dwarf *le* recessive | inflated *pp* or *vv* recessive | Axial *Fa* or *Fas* Dominant |  |
| Wadford's Unvergleichliche | Round *RR* Dominant | green *ii* recessive | white *aa* recessive | tall *Le* Dominant | Normal *PP* or *VV* Dominant | Axial *Fa* or *Fas* Dominant |  |
| Flack's Dwarf Imperial | Round *RR* Dominant | green *ii* recessive | white *aa* recessive | dwarf *le* recessive | inflated *pp* or *vv* recessive | Axial *Fa* or *Fas* Dominant |  |
| Frühe Englische Schwert Zuckererbse | wrinkled *rr* recessive | Yellow *II* Dominant | Purple-red *AA* Dominant | tall *Le* Dominant | inflated *pp* or *vv* recessive | Axial *Fa* or *Fas* Dominant | Green *Gp* Dominant |
| Zuckererbse, Weisse Schote | wrinkled *rr* recessive | Yellow *II* Dominant | Purple-red *AA* Dominant | tall *Le* Dominant | inflated *pp* or *vv* recessive | Axial *Fa* or *Fas* Dominant | white (yellow) g*p* recessive |
| Kronen Zuckererbse mit Weisser Schaale | wrinkled *rr* recessive | Yellow *II* Dominant | Purple-red *AA* Dominant | tall *Le* Dominant | inflated *pp* or *vv* recessive | terminal *fa* or f*as* recessive | white (yellow) g*p* recessive |
| Karr's Mammouth | wrinkled *rr* recessive | Yellow *II* Dominant | white *aa* recessive | tall *Le* Dominant | Normal *PP* or *VV* Dominant | Axial *Fa* or *Fas* Dominant |  |
| Nonpareil Knight's Marrow | wrinkled *rr* recessive | green *ii* recessive | white *aa* recessive | tall *Le* Dominant | Normal *PP* or *VV* Dominant | Axial *Fa* or *Fas* Dominant |  |

The names of varieties listed by Bouché (1854) are given for those where the status of several Mendelian characters can be determined. The full list can be found in the online version of this publication available at: https://bit.ly/2Zor5V2. The inferred genotype is given based on the gene assignments given in Ellis et al. (2011). The status of green vs yellow pod colour (*Gp* vs *gp*) was not listed for all varieties, but it is likely that those not designated as yellow or white podded were green podded (*Gp*). None of the varieties were designated as purple podded - one character which Mendel noted, but for which he did not present any data. Although the expected flowering time was given for some of these varieties, it is difficult to assign a corresponding genotype based on this information alone

## Supplementary Table 1.2 Characters that would segregate in the F2 of crosses between selected varieties from Bouché (1854)

|  | **Variety** | 1 | 2 | 3 | 4 | 5 | 6 | 7 | 8 | 9 | 10 | 11 | 12 | 14 | 15 | 16 |
| --- | --- | --- | --- | --- | --- | --- | --- | --- | --- | --- | --- | --- | --- | --- | --- | --- |
| 1 | Holländische Schifferbse |  | *A/a* | *A/a* |  |  |  |  |  |  |  |  |  |  |  |  |
| 2 | Thurnston's Reliance | 1 |  |  | *Fa*/*fa* or *Fas*/*fas* |  | *P*/*p* or *Vs*/*v* | *Le/le* |  | *I/i* |  |  |  |  | *R/r* |  |
| 3 | Auvergne-Erbse | 1 | 0 |  | *Fa*/*fa* or *Fas*/*fas* | *P*/*p* or *Vs*/*v* | *P*/*p* or *Vs*/*v* | *Le/le* |  | *I/i* |  |  |  |  | *R/r* |  |
| 4 | Dolden-Erbse, Mummie-Erbse, Türkische-Erbse, Kronen-Erbse | 2 | 1 | 1 |  |  |  |  |  |  |  |  |  |  |  |  |
| 5 | Zuckerschote mit gelben Huelsen | 2 | 2 | 1 | 2 |  |  |  | *Le/le* |  |  |  |  |  |  |  |
| 6 | Weisse Schwert Zuckererbse | 2 | 1 | 1 | 2 | 0 |  |  | *Le/le* |  |  |  |  |  |  |  |
| 7 | Bishop Longpod | 2 | 1 | 1 | 2 | 2 | 2 |  | *P*/*p* or *V*/*v* |  |  |  |  |  |  |  |
| 8 | Waite's Queen of Dwarfs | 3 | 2 | 2 | 3 | 1 | 1 | 1 |  |  | *I/i* |  |  |  |  |  |
| 9 | Wadford's Unvergleichliche | 2 | 1 | 1 | 2 | 2 | 2 | 2 | 3 |  |  |  |  |  |  | *R/r* |
| 10 | Flack's Dwarf Imperial | 4 | 3 | 3 | 4 | 2 | 2 | 2 | 1 | 2 |  |  |  |  |  |  |
| 11 | Frühe Englische Schwert Zuckererbse | 2 | 3 | 3 | 4 | 3 | 2 | 4 | 3 | 4 | 4 |  | *Gp/gp* |  |  |  |
| 12 | Zuckererbse, Weisse Schote | 2 | 4 | 3 | 4 | 2 | 2 | 4 | 3 | 4 | 4 | 1 |  | *Fa*/*fa* or *Fas*/*fas* |  |  |
| 13 | Kronen Zuckererbse mit Weisser Schaale | 3 | 5 | 4 | 3 | 3 | 3 | 5 | 4 | 5 | 5 | 2 | 1 |  |  |  |
| 14 | Karr's Mammouth | 2 | 1 | 1 | 2 | 2 | 2 | 2 | 3 | 2 | 4 | 2 | 2 | 3 |  | *I* vs *i* |
| 15 | Nonpareil Knight's Marrow | 3 | 2 | 2 | 3 | 3 | 3 | 3 | 4 | 1 | 3 | 3 | 3 | 4 | 1 |  |

The table indicates the number Mendelian characters that would segregate in the F2 of crosses between the varieties in Supplementary Table 1.1. For all 156 pairwise combinations the number of Mendelian characters that would segregate in the F2 of a cross is given in the lower triangle. For those crosses where there is a single differentiating character, the corresponding gene is indicated in the upper triangle. Three varieties were noted as yellow (or white) podded (*gpgp* shaded in yellow), but only two were designated as green podded (*Gp* shaded in green). It is most likely that the remaining varieties were also green podded (*Gp*). For crosses between the three yellow podded varieties and those not noted as green podded, one additional character may segregate, the cells where this applies are highlighted in yellow. If there is an additional character segregating in these cases then two of the crosses noted as segregating for one character would, in addition, segregate for *Gp/gp* and 1 cross that is monomorphic would segregate for *Gp/gp,* the corresponding cells are highlighted in purple. Note that undoubtedly, other character differences between these varieties would have existed; this table refers only to characters noted by Bouché (1854) for which Mendel studied their segregation.

## Supplementary Table 1.3. Mendel's segregation ratios (modified after Edwards 1986a)

In this table the observed and expected ratios of segregants in Mendel's experiments are presented. The page where the data can be found in the BSHS translation of Mendel's paper is indicated in the column marked §. A similar table was presented by Edwards (1986a, 2008) and the row number in that table is indicated in the column marked ¶.

This table differs from Edwards' table in several ways. The first concerns Mendel's first and second experiments and how these data are aggregated or disaggregated. For both these experiments the data are presented (i) for the experiment as a whole, (ii) for the experiment with the data for the first ten F1 plants removed and treated separately, and (iii) for the experiment with the first ten plants and the two extremes removed and treated separately. These three treatments generate data sets of 88, 84 and 64 χ and χ^2^ values. The sign of the χ values is according to Edwards (1986a).

Further to this there is a question as to which ratio should be used for the F3 data of the plant characters and for anthocyanin segregation in the trifactorial cross. The data is analysed without these data, with these data assuming Fishers ratio and with these data assuming a 2:1 segregation ratio. The corresponding statistics are at the bottom of the table.

There is a minor discrepancy between the data presented by here and in the BSHS translation of Mendel's translation of Mendel's paper for the first experiment on the reproductive cells. The difference is indicated in the table.

The analyses by Fisher (1936) and Pires and Branco (2010) are more complex and involves a variety of degrees of freedom. Here we have followed Edwards' helpful way of analysing the data as a set of ratios with one degree of freedom.

|  |  |  |  | **Expected** |  |  |  |  | **Observed** | |  |  |  |  |  |  |  |
| --- | --- | --- | --- | --- | --- | --- | --- | --- | --- | --- | --- | --- | --- | --- | --- | --- | --- |
|  | § | ¶ |  | **Ratio** |  | **Number** |  |  | **Number** |  | **total** |  | **χ2** | **χ** |  | **Comments** |  |
| **1) First and second experiments, all data i.e. Mendel's values including the 10 examples and 2 extremes for each** | | | | | | | | | | | | | | |  |  |  |
|  |  |  |  |  |  | ***R_*** | ***rr*** |  | ***R_*** | ***rr*** |  |  |  |  |  |  |  |
|  | 12 | *1* |  | 3:1 |  | 5,493.00 | 1,831.00 |  | 5474 | 1850 | 7324 |  | 0.262880 | -0.512718 |  | *R_:rr* |  |
|  |  |  |  |  |  | ***I_*** | ***ii*** |  | ***I_*** | ***ii*** |  |  |  |  |  |  |  |
|  | 12 | *2* |  | 3:1 |  | 6,017.25 | 2,005.75 |  | 6022 | 2001 | 8023 |  | 0.014999 | 0.122469 |  | *I_:ii* |  |
|  |  |  |  |  |  |  |  |  |  |  |  |  |  |  |  |  |  |
| **2) First and second experiments, as Edwards, including extreme segregations, but excluding the 10 examples.** | | | | | | | | | | | | | | |  |  |  |
|  |  |  |  |  |  | ***R_*** | ***rr*** |  | ***R_*** | ***rr*** |  |  |  |  |  |  |  |
|  | 12 | *1* |  | 3:1 |  | 5,165.25 | 1,721.75 |  | 5138 | 1749 | 6887 |  | 0.575045 | -0.758317 |  | *R_:rr* |  |
|  |  |  |  |  |  | ***I_*** | ***ii*** |  | ***I_*** | ***ii*** |  |  |  |  |  |  |  |
|  | 12 | *2* |  | 3:1 |  | 5,658.75 | 1,886.25 |  | 5667 | 1878 | 7545 |  | 0.048111 | 0.219343 |  | *I_:ii* |  |
|  |  |  |  |  |  |  |  |  |  |  |  |  |  |  |  |  |  |
| **3) First and second experiments, all data excluding the 10 examples and two extremes for each experiment** | | | | | | | | | | | | | | |  |  |  |
|  |  |  |  |  |  | ***R_*** | ***rr*** |  | ***R_*** | ***rr*** |  |  |  |  |  |  |  |
|  | 12 | *1* |  | 3:1 |  | 5,109.75 | 1,703.25 |  | 5081 | 1732 | 6813 |  | 0.647047 | -0.804393 |  | *R_:rr* |  |
|  |  |  |  |  |  | ***I_*** | ***ii*** |  | ***I_*** | ***ii*** |  |  |  |  |  |  |  |
|  | 12 | *2* |  | 3:1 |  | 5,604.75 | 1,868.25 |  | 5615 | 1858 | 7473 |  | 0.074981 | 0.273827 |  | *I_:ii* |  |
|  |  |  |  |  |  |  |  |  |  |  |  |  |  |  |  |  |  |
| **4) First experiment, F2 from first 10 F1 plants** | | | | | | | | | |  |  |  |  |  |  |  |  |
|  |  |  |  |  |  | ***R_*** | ***rr*** |  | ***R_*** | ***rr*** |  |  |  |  |  |  |  |
|  | 13 | 3 |  | 3:1 |  | 42.75 | 14.25 |  | 45 | 12 | 57 |  | 0.473684 | 0.688247 |  | *R_:rr* | plant#1 |
|  | 13 | 4 |  | 3:1 |  | 26.25 | 8.75 |  | 27 | 8 | 35 |  | 0.085714 | 0.292770 |  | *R_:rr* | plant#2 |
|  | 13 | 5 |  | 3:1 |  | 23.25 | 7.75 |  | 24 | 7 | 31 |  | 0.096774 | 0.311086 |  | *R_:rr* | plant#3 |
|  | 13 | 6 |  | 3:1 |  | 21.75 | 7.25 |  | 19 | 10 | 29 |  | 1.390805 | -1.179324 |  | *R_:rr* | plant#4 |
|  | 13 | 7 |  | 3:1 |  | 32.25 | 10.75 |  | 32 | 11 | 43 |  | 0.007752 | -0.088045 |  | *R_:rr* | plant#5 |
|  | 13 | 8 |  | 3:1 |  | 24.00 | 8.00 |  | 26 | 6 | 32 |  | 0.666667 | 0.816497 |  | *R_:rr* | plant#6 |
|  | 13 | 9 |  | 3:1 |  | 84.00 | 28.00 |  | 88 | 24 | 112 |  | 0.761905 | 0.872872 |  | *R_:rr* | plant#7 |
|  | 13 | 10 |  | 3:1 |  | 24.00 | 8.00 |  | 22 | 10 | 32 |  | 0.666667 | -0.816497 |  | *R_:rr* | plant#8 |
|  | 13 | 11 |  | 3:1 |  | 25.50 | 8.50 |  | 28 | 6 | 34 |  | 0.980392 | 0.990148 |  | *R_:rr* | plant#9 |
|  | 13 | 12 |  | 3:1 |  | 24.00 | 8.00 |  | 25 | 7 | 32 |  | 0.166667 | 0.408248 |  | *R_:rr* | plant#10 |
|  |  |  |  |  |  |  |  |  |  |  |  |  |  |  |  |  |  |
| **5) Second experiment, F2 from first 10 F1 plants** | | | | | | | |  |  |  |  |  |  |  |  |  |  |
|  |  |  |  |  |  | ***I_*** | ***ii*** |  | ***I_*** | ***ii*** |  |  |  |  |  |  |  |
|  | 13 | 13 |  | 3:1 |  | 27.00 | 9.00 |  | 25 | 11 | 36 |  | 0.592593 | -0.769800 |  | *I_:ii* | plant#1 |
|  | 13 | 14 |  | 3:1 |  | 29.25 | 9.75 |  | 32 | 7 | 39 |  | 1.034188 | 1.016950 |  | *I_:ii* | plant#2 |
|  | 13 | 15 |  | 3:1 |  | 14.25 | 4.75 |  | 14 | 5 | 19 |  | 0.017544 | -0.132453 |  | *I_:ii* | plant#3 |
|  | 13 | 16 |  | 3:1 |  | 72.75 | 24.25 |  | 70 | 27 | 97 |  | 0.415808 | -0.644831 |  | *I_:ii* | plant#4 |
|  | 13 | 17 |  | 3:1 |  | 27.75 | 9.25 |  | 24 | 13 | 37 |  | 2.027027 | -1.423737 |  | *I_:ii* | plant#5 |
|  | 13 | 18 |  | 3:1 |  | 19.50 | 6.50 |  | 20 | 6 | 26 |  | 0.051282 | 0.226455 |  | *I_:ii* | plant#6 |
|  | 13 | 19 |  | 3:1 |  | 33.75 | 11.25 |  | 32 | 13 | 45 |  | 0.362963 | -0.602464 |  | *I_:ii* | plant#7 |
|  | 13 | 20 |  | 3:1 |  | 39.75 | 13.25 |  | 44 | 9 | 53 |  | 1.817610 | 1.348188 |  | *I_:ii* | plant#8 |
|  | 13 | 21 |  | 3:1 |  | 48.00 | 16.00 |  | 50 | 14 | 64 |  | 0.333333 | 0.577350 |  | *I_:ii* | plant#9 |
|  | 13 | 22 |  | 3:1 |  | 46.50 | 15.50 |  | 44 | 18 | 62 |  | 0.537634 | -0.733236 |  | *I_:ii* | plant#10 |
|  |  |  |  |  |  |  |  |  |  |  |  |  |  |  |  |  |  |
| **6) First experiment, extreme examples given in the text** | | | | | | | |  |  |  |  |  |  |  |  |  |  |
|  |  |  |  |  |  | ***R_*** | ***rr*** |  | ***R_*** | ***rr*** |  |  |  |  |  |  |  |
|  | 13 |  |  | 3:1 |  | 33.75 | 11.25 |  | 43 | 2 | 45 |  | 10.140741 | 3.184453 |  | *R_:rr* | notinEdwards |
|  | 13 |  |  | 3:1 |  | 21.75 | 7.25 |  | 14 | 15 | 29 |  | 11.045977 | -3.323549 |  | *R_:rr* | notinEdwards |
|  |  |  |  |  |  |  |  |  |  |  |  |  |  |  |  |  |  |
| **7) Second experiment, extreme examples given in the text** | | | | | | | |  |  |  |  |  |  |  |  |  |  |
|  |  |  |  |  |  | ***I_*** | ***ii*** |  | ***I_*** | ***ii*** |  |  |  |  |  |  |  |
|  | 13 |  |  | 3:1 |  | 24.75 | 8.25 |  | 32 | 1 | 33 |  | 8.494949 | 2.914610 |  | *I_:ii* | notinEdwards |
|  | 13 |  |  | 3:1 |  | 29.25 | 9.75 |  | 20 | 19 | 39 |  | 11.700855 | -3.420651 |  | *I_:ii* | notinEdwards |
|  |  |  |  |  |  |  |  |  |  |  |  |  |  |  |  |  |  |
| **8) F2 analysis experiments 3 to 7** | | | | |  |  |  |  |  |  |  |  |  |  |  |  |  |
|  |  |  |  |  |  | ***X_*** | ***xx*** |  | ***X_*** | ***xx*** |  |  |  |  |  |  |  |
|  | 14 | 23 |  | 3:1 |  | 696.75 | 232.25 |  | 705 | 224 | 929 |  | 0.390743 | 0.625094 |  | *A_:aa* |  |
|  | 14 | 24 |  | 3:1 |  | 885.75 | 295.25 |  | 882 | 299 | 1181 |  | 0.063506 | -0.252003 |  | *P_:pp* | or*V_:vv* |
|  | 14 | 25 |  | 3:1 |  | 435.00 | 145.00 |  | 428 | 152 | 580 |  | 0.450575 | -0.671249 |  | *Gp_:gpgp* |  |
|  | 14 | 26 |  | 3:1 |  | 643.50 | 214.50 |  | 651 | 207 | 858 |  | 0.349650 | 0.591312 |  | *Fa_:fafa* | or*Fas_:fasfas* |
|  | 14 | 27 |  | 3:1 |  | 798.00 | 266.00 |  | 787 | 277 | 1064 |  | 0.606516 | -0.778792 |  | *Le_:lele* |  |
|  |  |  |  |  |  |  |  |  |  |  |  |  |  |  |  |  |  |
| **9) F2:F3 experiment, seed characters (bulk analysis)** | | | | | | | |  |  |  |  |  |  |  |  |  |  |
|  |  |  |  |  |  | ***Xx*** | ***XX*** |  | ***Xx*** | ***XX*** |  |  |  |  |  |  |  |
|  | 15 | 28 |  | 2:1 |  | 376.67 | 188.33 |  | 372 | 193 | 565 |  | 0.173451 | -0.416475 |  | *Rr:RR* |  |
|  | 15 | 29 |  | 2:1 |  | 346.00 | 173.00 |  | 353 | 166 | 519 |  | 0.424855 | 0.651809 |  | *Ii:II* |  |
|  |  |  |  |  |  |  |  |  |  |  |  |  |  |  |  |  |  |
| **10) F2:F3 experiment, plant characters (10 F3 plants from 100 F2s)** | | | | | | | | | |  |  |  |  |  |  |  |  |
|  |  |  |  |  |  | ***Xx*** | ***XX*** |  | ***Xx*** | ***XX*** |  |  |  |  |  |  |  |
|  | 16 | 30 |  | 1.8874:1.1126 |  | 62.91 | 37.09 |  | 64 | 36 | 100 |  | 0.050693 | 0.225151 |  | *Aa:AA* |  |
|  | 16 | 31 |  | 1.8874:1.1126 |  | 62.91 | 37.09 |  | 71 | 29 | 100 |  | 2.803309 | 1.674309 |  | *Pp:PP* | or*Vv:VV* |
|  | 16 | 32 |  | 1.8874:1.1126 |  | 62.91 | 37.09 |  | 60 | 40 | 100 |  | 0.363536 | -0.602939 |  | *Gpgp:GpGp* |  |
|  | 16 | 33 |  | 1.8874:1.1126 |  | 62.91 | 37.09 |  | 67 | 33 | 100 |  | 0.716086 | 0.846219 |  | *Fafa:FaFa* | or *Fasfas:FasFas* |
|  | 16 | 34 |  | 1.8874:1.1126 |  | 62.91 | 37.09 |  | 72 | 28 | 100 |  | 3.539407 | 1.881331 |  | *Lele:LeLe* |  |
|  | 16 | 35 |  | 1.8874:1.1126 |  | 62.91 | 37.09 |  | 65 | 35 | 100 |  | 0.186774 | 0.432174 |  | *Gpgp:GpGp* |  |
|  |  |  |  |  |  |  |  |  |  |  |  |  |  |  |  |  |  |
| **11) F2:F3 experiment, plant characters (not assuming exactly 10 F3 plants from 100 F2s)** | | | | | | | | | | | |  |  |  |  |  |  |
|  |  |  |  |  |  | ***Xx*** | ***XX*** |  | ***Xx*** | ***XX*** |  |  |  |  |  |  |  |
|  | 16 | 1 |  | 2:1 |  | 66.67 | 33.33 |  | 64 | 36 | 100 |  | 0.320000 | -0.565685 |  | *Aa:AA* |  |
|  | 16 | 2 |  | 2:1 |  | 66.67 | 33.33 |  | 71 | 29 | 100 |  | 0.845000 | 0.919239 |  | *Pp:PP* | or*Vv:VV* |
|  | 16 | 3 |  | 2:1 |  | 66.67 | 33.33 |  | 60 | 40 | 100 |  | 2.000000 | -1.414214 |  | *Gpgp:GpGp* |  |
|  | 16 | 4 |  | 2:1 |  | 66.67 | 33.33 |  | 67 | 33 | 100 |  | 0.005000 | 0.070711 |  | *Fafa:FaFa* | or *Fasfas:FasFas* |
|  | 16 | 5 |  | 2:1 |  | 66.67 | 33.33 |  | 72 | 28 | 100 |  | 1.280000 | 1.131371 |  | *Lele:LeLe* |  |
|  | 16 | 6 |  | 2:1 |  | 66.67 | 33.33 |  | 65 | 35 | 100 |  | 0.125000 | -0.353553 |  | *Gpgp:GpGp* |  |
|  |  |  |  |  |  |  |  |  |  |  |  |  |  |  |  |  |  |
| **12) Bifactorial cross F2** | | | | |  |  |  |  |  |  |  |  |  |  |  |  |  |
|  |  |  |  |  |  | ***X_*** | ***xx*** |  | ***X_*** | ***xx*** |  |  |  |  |  |  | subclass |
|  | 19 | 36 |  | 3:1 |  | 417.00 | 139.00 |  | 423 | 133 | 556 |  | 0.345324 | 0.587643 |  | *R_:rr* | all |
|  | 19 | 37 |  | 3:1 |  | 317.25 | 105.75 |  | 315 | 108 | 423 |  | 0.063830 | -0.252646 |  | *Ii:II* | *RR* |
|  | 19 | 38 |  | 3:1 |  | 99.75 | 33.25 |  | 101 | 32 | 133 |  | 0.062657 | 0.250313 |  | *Ii:II* | *rr* |
|  |  |  |  |  |  |  |  |  |  |  |  |  |  |  |  |  |  |
| **13) Bifactorial cross F3** | | | | |  |  |  |  |  |  |  |  |  |  |  |  |  |
|  | 19 | 39 |  | 2:1 |  | 200.67 | 100.33 |  | 198 | 103 | 301 |  | 0.106312 | -0.326056 |  | *Rr:RR* | *R_I_* |
|  | 19 | 40 |  | 2:1 |  | 68.00 | 34.00 |  | 67 | 35 | 102 |  | 0.044118 | -0.210042 |  | *Rr:RR* | *R_ii* |
|  | 19 | 41 |  | 2:1 |  | 64.00 | 32.00 |  | 68 | 28 | 96 |  | 0.750000 | 0.866025 |  | *Ii:II* | *rrI_* |
|  | 19 | 42 |  | 2:1 |  | 132.00 | 66.00 |  | 138 | 60 | 198 |  | 0.818182 | 0.904534 |  | *Ii:II* | *RrI_* |
|  | 19 | 43 |  | 2:1 |  | 68.67 | 34.33 |  | 65 | 38 | 103 |  | 0.587379 | -0.766406 |  | *Ii:II* | *RRI_* |
|  |  |  |  |  |  |  |  |  |  |  |  |  |  |  |  |  |  |
| **14) Trifactorial cross F2** | | | | |  |  |  |  |  |  |  |  |  |  |  |  |  |
|  |  |  |  |  |  | ***X_*** | ***xx*** |  | ***X_*** | ***xx*** |  |  |  |  |  |  | subclass |
|  | 21 | 44 |  | 3:1 |  | 479.25 | 159.75 |  | 480 | 159 | 639 |  | 0.004695 | 0.068519 |  | *R_:rr* | all |
|  | 21 | 45 |  | 3:1 |  | 360.00 | 120.00 |  | 367 | 113 | 480 |  | 0.544444 | 0.737865 |  | *I_:ii* | *R_* |
|  | 21 | 46 |  | 3:1 |  | 119.25 | 39.75 |  | 122 | 37 | 159 |  | 0.253669 | 0.503655 |  | *I_:ii* | *rr* |
|  |  |  |  |  |  |  |  |  |  |  |  |  |  |  |  |  |  |
| **15) Trifactorial cross F3** | | | | |  |  |  |  |  |  |  |  |  |  |  |  |  |
|  |  |  |  |  |  | ***Xx*** | ***XX*** |  | ***Xx*** | ***XX*** |  |  |  |  |  |  | subclass |
|  | 21 | 47 |  | 2:1 |  | 244.67 | 122.33 |  | 245 | 122 | 367 |  | 0.001362 | 0.036911 |  | *Rr:RR* | *R_I_* |
|  | 21 | 48 |  | 2:1 |  | 75.33 | 37.67 |  | 76 | 37 | 113 |  | 0.017699 | 0.133038 |  | *Rr:RR* | *R_ii* |
|  | 21 | 49 |  | 2:1 |  | 81.33 | 40.67 |  | 79 | 43 | 122 |  | 0.200820 | -0.448129 |  | *Ii:II* | *rrI_* |
|  | 21 | 50 |  | 2:1 |  | 163.33 | 81.67 |  | 175 | 70 | 245 |  | 2.500000 | 1.581139 |  | *Ii:II* | *RrI_* |
|  | 21 | 51 |  | 2:1 |  | 81.33 | 40.67 |  | 78 | 44 | 122 |  | 0.409836 | -0.640184 |  | *Ii:II* | *RRI_* |
|  |  |  |  |  |  |  |  |  |  |  |  |  |  |  |  |  |  |
| **16) Tri-factorial cross F2, Anthocyanin pigmentation** | | | | | | | |  |  |  |  |  |  |  |  |  |  |
|  |  |  |  |  |  | ***X_*** | ***xx*** |  | ***X_*** | ***xx*** |  |  |  |  |  |  | subclass |
|  | 21 | 52 |  | 3:1 |  | 131.25 | 43.75 |  | 127 | 48 | 175 |  | 0.550476 | -0.741941 |  | *A_:aa* | *RrIi* |
|  | 21 | 53 |  | 3:1 |  | 52.50 | 17.50 |  | 52 | 18 | 70 |  | 0.019048 | -0.138013 |  | *A_:aa* | *RrII* |
|  | 21 | 54 |  | 3:1 |  | 58.50 | 19.50 |  | 60 | 18 | 78 |  | 0.153846 | 0.392232 |  | *A_:aa* | *RRIi* |
|  | 21 | 55 |  | 3:1 |  | 33.00 | 11.00 |  | 30 | 14 | 44 |  | 1.090909 | -1.044466 |  | *A_:aa* | *RRII* |
|  | 21 | 56 |  | 3:1 |  | 57.00 | 19.00 |  | 60 | 16 | 76 |  | 0.631579 | 0.794719 |  | *A_:aa* | *Rrii* |
|  | 21 | 57 |  | 3:1 |  | 27.75 | 9.25 |  | 26 | 11 | 37 |  | 0.441441 | -0.664411 |  | *A_:aa* | *RRii* |
|  | 21 | 58 |  | 3:1 |  | 59.25 | 19.75 |  | 55 | 24 | 79 |  | 1.219409 | -1.104269 |  | *A_:aa* | *rrIi* |
|  | 21 | 59 |  | 3:1 |  | 32.25 | 10.75 |  | 33 | 10 | 43 |  | 0.069767 | 0.264135 |  | *A_:aa* | *rrII* |
|  | 21 | 60 |  | 3:1 |  | 27.75 | 9.25 |  | 30 | 7 | 37 |  | 0.729730 | 0.854242 |  | *A_:aa* | *rrii* |
|  |  |  |  |  |  |  |  |  |  |  |  |  |  |  |  |  |  |
| **17) Tri-factorial cross F3 Anthocyanin pigmentation (Fisher's ratio)** | | | | | | | | | |  |  |  |  |  |  |  |  |
|  |  |  |  |  |  | ***Xx*** | ***XX*** |  | ***Xx*** | ***XX*** |  |  |  |  |  |  | subclass |
|  | 21 | 61 |  | 1.8874:1.1126 |  | 79.90 | 47.10 |  | 78 | 49 | 127 |  | 0.121670 | -0.348813 |  | *Aa:AA* | *RrIi* |
|  | 21 | 62 |  | 1.8874:1.1126 |  | 32.71 | 19.29 |  | 38 | 14 | 52 |  | 2.302554 | 1.517417 |  | *Aa:AA* | *RrII* |
|  | 21 | 63 |  | 1.8874:1.1126 |  | 37.75 | 22.25 |  | 45 | 15 | 60 |  | 3.757200 | 1.938350 |  | *Aa:AA* | *RRIi* |
|  | 21 | 64 |  | 1.8874:1.1126 |  | 18.87 | 11.13 |  | 22 | 8 | 30 |  | 1.396262 | 1.181635 |  | *Aa:AA* | *RRII* |
|  | 21 | 65 |  | 1.8874:1.1126 |  | 37.75 | 22.25 |  | 40 | 20 | 60 |  | 0.362434 | 0.602025 |  | *Aa:AA* | *Rrii* |
|  | 21 | 66 |  | 1.8874:1.1126 |  | 16.36 | 9.64 |  | 17 | 9 | 26 |  | 0.068104 | 0.260967 |  | *Aa:AA* | *RRii* |
|  | 21 | 67 |  | 1.8874:1.1126 |  | 34.60 | 20.40 |  | 36 | 19 | 55 |  | 0.152331 | 0.390296 |  | *Aa:AA* | *rrIi* |
|  | 21 | 68 |  | 1.8874:1.1126 |  | 20.76 | 12.24 |  | 25 | 8 | 33 |  | 2.333603 | 1.527614 |  | *Aa:AA* | *rrII* |
|  | 21 | 69 |  | 1.8874:1.1126 |  | 18.87 | 11.13 |  | 20 | 10 | 30 |  | 0.181217 | 0.425696 |  | *Aa:AA* | *rrii* |
| **18) Tri-factorial cross F3 Anthocyanin pigmentation assuming 2:1 segregation** | | | | | | | | | | |  |  |  |  |  |  |  |
|  |  |  |  |  |  | ***Xx*** | ***XX*** |  | ***Xx*** | ***XX*** |  |  |  |  |  |  | subclass |
|  | 21 | 1 |  | 2:1 |  | 84.67 | 42.33 |  | 78 | 49 | 127 |  | 1.574803 | -1.254912 |  | *Aa:AA* | *RrIi* |
|  | 21 | 2 |  | 2:1 |  | 34.67 | 17.33 |  | 38 | 14 | 52 |  | 0.961538 | 0.980581 |  | *Aa:AA* | *RrII* |
|  | 21 | 3 |  | 2:1 |  | 40.00 | 20.00 |  | 45 | 15 | 60 |  | 1.875000 | 1.369306 |  | *Aa:AA* | *RRIi* |
|  | 21 | 4 |  | 2:1 |  | 20.00 | 10.00 |  | 22 | 8 | 30 |  | 0.600000 | 0.774597 |  | *Aa:AA* | *RRII* |
|  | 21 | 5 |  | 2:1 |  | 40.00 | 20.00 |  | 40 | 20 | 60 |  | 0.000000 | 0.000000 |  | *Aa:AA* | *Rrii* |
|  | 21 | 6 |  | 2:1 |  | 17.33 | 8.67 |  | 17 | 9 | 26 |  | 0.019231 | -0.138675 |  | *Aa:AA* | *RRii* |
|  | 21 | 7 |  | 2:1 |  | 36.67 | 18.33 |  | 36 | 19 | 55 |  | 0.036364 | -0.190693 |  | *Aa:AA* | *rrIi* |
|  | 21 | 8 |  | 2:1 |  | 22.00 | 11.00 |  | 25 | 8 | 33 |  | 1.227273 | 1.107823 |  | *Aa:AA* | *rrII* |
|  | 21 | 9 |  | 2:1 |  | 20.00 | 10.00 |  | 20 | 10 | 30 |  | 0.000000 | 0.000000 |  | *Aa:AA* | *rrii* |
|  |  |  |  |  |  |  |  |  |  |  |  |  |  |  |  |  |  |
| **19) Reproductive cells, experiment 1** | | | | | | |  |  |  |  |  |  |  |  |  |  |  |
|  |  |  |  |  |  | ***Xx*** | ***XX*** |  | ***Xx*** | ***XX*** |  |  |  |  |  |  | subclass |
|  | 27 | 70 |  | 1:1 |  | 45.00 | 45.00 |  | 47 ^a^ | 43 | 90 |  | 0.177778 | -0.421637 |  | *Rr:RR* | all |
|  | 27 | 71 |  | 1:1 |  | 21.50 | 21.50 |  | 23 | 20 | 43 |  | 0.209302 | -0.457496 |  | *Ii:II* | *RR* |
|  | 27 | 72 |  | 1:1 |  | 23.50 | 23.50 |  | 22 ^a^ | 25 | 47 |  | 0.191489 | 0.437595 |  | *Ii:II* | *Rr* |
|  | | | | |  |  |  |  | ^a^47 and 22 are 45 and 20 respectively in the BSHS translation | | | | | | | | |
|  | | | | |  |  |  |  |  | | | | | | | | |
| **20) Reproductive cells, experiment 2** | | | | | | |  |  |  |  |  |  |  |  |  |  | subclass |
|  |  |  |  |  |  | ***Xx*** | ***xx*** |  | ***Xx*** | ***xx*** |  |  |  |  |  |  | subclass |
|  | 27 | 73 |  | 1:1 |  | 55.00 | 55.00 |  | 57 | 53 | 110 |  | 0.145455 | 0.381385 |  | *Rr:rr* | all |
|  | 27 | 74 |  | 1:1 |  | 28.50 | 28.50 |  | 31 | 26 | 57 |  | 0.438596 | 0.662266 |  | *Ii:ii* | *Rr* |
|  | 27 | 75 |  | 1:1 |  | 26.50 | 26.50 |  | 27 | 26 | 53 |  | 0.018868 | 0.137361 |  | *Ii:ii* | *rr* |
| **21) Reproductive cells, experiment 3** | | | | | | |  |  |  |  |  |  |  |  |  |  |  |
|  |  |  |  |  |  | ***Xx*** | ***XX*** |  | ***Xx*** | ***XX*** |  |  |  |  |  |  | subclass |
|  | 27 | 76 |  | 1:1 |  | 43.50 | 43.50 |  | 43 | 44 | 87 |  | 0.011494 | 0.107211 |  | *Rr:RR* | all |
|  | 27 | 77 |  | 1:1 |  | 22.00 | 22.00 |  | 19 | 25 | 44 |  | 0.818182 | 0.904534 |  | *Ii:II* | *RR* |
|  | 27 | 78 |  | 1:1 |  | 21.50 | 21.50 |  | 21 | 22 | 43 |  | 0.023256 | 0.152499 |  | *Ii:II* | *Rr* |
|  | | | | |  |  |  |  |  |  |  |  |  |  |  |  |  |
| **22) Reproductive cells, experiment 4** | | | | | | |  |  |  |  |  |  |  |  |  |  | subclass |
|  |  |  |  |  |  | ***Xx*** | ***xx*** |  | ***Xx*** | ***xx*** |  |  |  |  |  |  | subclass |
|  | 27 | 79 |  | 1:1 |  | 49.00 | 49.00 |  | 49 | 49 | 98 |  | 0.000000 | 0.000000 |  | *Rr:rr* | all |
|  | 27 | 80 |  | 1:1 |  | 24.50 | 24.50 |  | 24 | 25 | 49 |  | 0.020408 | -0.142857 |  | *Ii:ii* | *Rr* |
|  | 27 | 81 |  | 1:1 |  | 24.50 | 24.50 |  | 22 | 27 | 49 |  | 0.510204 | -0.714286 |  | *Ii:ii* | *rr* |
|  | | | | |  |  |  |  |  |  |  |  |  |  |  |  |  |
| **23) Reproductive cells, plant characters** | | | | | | | |  |  |  |  |  |  |  |  |  | subclass |
|  |  |  |  |  |  | ***Xx*** | ***xx*** |  | ***Xx*** | ***xx*** |  |  |  |  |  |  | subclass |
|  | 28 | 82 |  | 1:1 |  | 83.00 | 83.00 |  | 87 | 79 | 166 |  | 0.385542 | 0.620920 |  | *Lele:lele* | all |
|  | 28 | 83 |  | 1:1 |  | 43.50 | 43.50 |  | 47 | 40 | 87 |  | 0.563218 | 0.750479 |  | *Aa:aa* | *Lele* |
|  | 28 | 84 |  | 1:1 |  | 39.50 | 39.50 |  | 38 | 41 | 79 |  | 0.113924 | -0.337526 |  | *Aa:aa* | *lele* |
|  | **§** |  | **page in Mendel manuscript at BSHS** | | | | |  |  |  |  | | |  |  |  |  |
|  |  | **¶** | **row number in Edwards (1986a,2008)** | | | | |  |  |  |  | | |  |  |  |  |
|  |  |  |  |  |  |  |  |  |  |  |  | | |  |  |  |  |

## Supplementary Table 1.4. χ and χ^2^ values for Mendel's data grouped in various ways

### Table 1.4a

In this table all of the ratios with one degree of freedom in Mendel's data are considered. For experiments 1 and 2 just one segregation ratio is considered - it is the aggregate of all the plants which includes the first 10 plants used as examples are and the two with extreme segregation ratios. For the F2:F3 experiment the segregation ratios for plant characters, and for anthocyanin pigmentation in the trifactorial cross, Fisher's ratio is assumed to be the expectation.

| **Mendel's Dataset** | | |  |  |  |
| --- | --- | --- | --- | --- | --- |
|  |  | **χ^2^** | **χ** |  |  |
|  |  | 64 | 64 |  | Count |
|  |  | 36.317 | 15.873 |  | Total |
|  |  | **0.5674** | **0.2480** |  | **Mean** |
|  |  | **0.6828** | **0.5140** |  | **Variance** |
|  |  | 0.8263 | 0.7169 |  | Std Dev |
|  |  | 3.7572 | 1.9383 |  | max |
|  |  | 0.0000 | -1.1043 |  | min |
|  |  |  |  |  |  |
|  |  |  | 22 |  | number -ve |
|  |  |  | 41 |  | number +ve |
|  |  |  | 1 |  | number =0 |

### Table 1.4b

In this table is as for 1.4a, but does not consider the segregation ratios for plant characters in the F2:F3 experiment or the anthocyanin pigmentation in the trifactorial cross.

| **Data excluding plant characters in the F3** | | | | | |
| --- | --- | --- | --- | --- | --- |
|  |  | **χ^2^** | **χ** |  |  |
|  |  | 49 | 49 |  | Count |
|  |  | 17.981 | 3.9216 |  | Total |
|  |  | **0.3670** | **0.0800** |  | **Mean** |
|  |  | **0.1862** | **0.3681** |  | **Variance** |
|  |  | 0.4315 | 0.6067 |  | Std Dev |
|  |  | 2.5000 | 1.5811 |  | max |
|  |  | 0.0000 | -1.1043 |  | min |
|  |  |  |  |  |  |
|  |  |  | 20 |  | number -ve |
|  |  |  | 28 |  | number +ve |
|  |  |  | 1 |  | number =0 |

### Table 1.4c

In this table is as for 1.4a, but assumes a 1:2 segregation ration for anthocyanin pigmentation in the trifactorial cross.

| **Mendel's Data set assuming 2:1 segregation** | | | | | |
| --- | --- | --- | --- | --- | --- |
| **for plant characters in the trifactorial expt** | | | | | |
|  |  | **χ^2^** | **χ** |  |  |
|  |  | 64 | 64 |  | Count |
|  |  | 31.935 | 11.026 |  | Total |
|  |  | **0.4990** | **0.1723** |  | **Mean** |
|  |  | **0.4676** | **0.4768** |  | **Variance** |
|  |  | 0.6838 | 0.6905 |  | Std Dev |
|  |  | 3.5394 | 1.8813 |  | max |
|  |  | 0.0000 | -1.2549 |  | min |
|  |  |  |  |  |  |
|  |  |  | 24 |  | number -ve |
|  |  |  | 37 |  | number +ve |
|  |  |  | 3 |  | number =0 |

### Table 1.4d

In this table the data for experiments 1 and 2 is fully disaggregated; the first ten plants used as examples and the two examples of extreme segregation ratios are treated separately and are not counted twice. For the F2:F3 experiment the segregation ratios for plant characters, and for anthocyanin pigmentation in the trifactorial cross, Fisher's ratio is assumed to be the expectation.

| **Complete Dataset** | | |  |  |  |
| --- | --- | --- | --- | --- | --- |
|  |  | **χ^2^** | **χ** |  |  |
|  |  | 88 | 88 |  | Count |
|  |  | 90.630 | 16.246 |  | Total |
|  |  | **1.0299** | **0.1846** |  | **Mean** |
|  |  | **4.8131** | **1.0073** |  | **Variance** |
|  |  | 2.1939 | 1.0036 |  | Std Dev |
|  |  | 11.701 | 3.1845 |  | max |
|  |  | 0.0000 | -3.4207 |  | min |
|  |  |  |  |  |  |
|  |  |  | 33 |  | number -ve |
|  |  |  | 54 |  | number +ve |
|  |  |  | 1 |  | number =0 |

### Table 1.4e

In this table the data are as in table 1.4d except that the segregation ratios for plant characters in the F2:F3 experiment and the anthocyanin pigmentation in the trifactorial cross are not considered.

| **Complete dataset** | | |  |  |  |
| --- | --- | --- | --- | --- | --- |
| **excluding plant characters in the F3** | | | | | |
|  |  | **χ^2^** | **χ** |  |  |
|  |  | 73 | 73 |  | Count |
|  |  | 72.295 | 4.2945 |  | Total |
|  |  | **0.9903** | **0.0588** |  | **Mean** |
|  |  | **5.4499** | **1.0006** |  | **Variance** |
|  |  | 2.3345 | 1.0003 |  | Std Dev |
|  |  | 11.701 | 3.1845 |  | max |
|  |  | 0.0000 | -3.4207 |  | min |
|  |  |  |  |  |  |
|  |  |  | 31 |  | number -ve |
|  |  |  | 41 |  | number +ve |
|  |  |  | 1 |  | number =0 |

### Table 1.4f

In this table the data are as in table 1.4d except that the segregation ratios for anthocyanin pigmentation in the trifactorial cross are not considered.

| **Complete data set assuming 2:1 segregation of plant** | | | | | |
| --- | --- | --- | --- | --- | --- |
| **characters in the F3 of the trifactorial experiment** | | | | | |
|  |  | **χ^2^** | **χ** |  |  |
|  |  | 88 | 88 |  | Count |
|  |  | 86.249 | 11.399 |  | Total |
|  |  | **0.9801** | **0.1295** |  | **Mean** |
|  |  | **4.7047** | **0.9744** |  | **Variance** |
|  |  | 2.1690 | 0.9871 |  | Std Dev |
|  |  | 11.701 | 3.1845 |  | max |
|  |  | 0.0000 | -3.4207 |  | min |
|  |  |  |  |  |  |
|  |  |  | 35 |  | number -ve |
|  |  |  | 50 |  | number +ve |
|  |  |  | 3 |  | number =0 |

### Table 1.4g

In this table the data are as in Edwards (1986a, 2008) except that the values for the reproductive cells, experiment 1 are as in the BSHS translation.

| **Edwards' Dataset** | | |  |  |  |
| --- | --- | --- | --- | --- | --- |
|  |  | **χ^2^** | **χ** |  |  |
|  |  | 84 | 84 |  | Count |
|  |  | 49.149 | 16.883 |  | Total |
|  |  | **0.5851** | **0.2010** |  | **Mean** |
|  |  | **0.5941** | **0.5513** |  | **Variance** |
|  |  | 0.7708 | 0.7425 |  | Std Dev |
|  |  | 3.7572 | 1.9383 |  | max |
|  |  | 0.0000 | -1.4237 |  | min |
|  |  |  |  |  |  |
|  |  |  | 31 |  | number -ve |
|  |  |  | 52 |  | number +ve |
|  |  |  | 1 |  | number =0 |

### Table 1.4h

In this table the data are as in table 1.4g except that the segregation ratios for plant characters in the F2:F3 experiment and the anthocyanin pigmentation in the trifactorial cross are not considered.

| **Edwards' Dataset** | | |  |  |  |
| --- | --- | --- | --- | --- | --- |
| **excluding plant characters in the F3** | | | | | |
|  |  | **χ^2^** | **χ** |  |  |
|  |  | 69 | 69 |  | Count |
|  |  | 30.814 | 4.9313 |  | Total |
|  |  | **0.4466** | **0.0715** |  | **Mean** |
|  |  | **0.2385** | **0.4480** |  | **Variance** |
|  |  | 0.4883 | 0.6693 |  | Std Dev |
|  |  | 2.5000 | 1.5811 |  | max |
|  |  | 0.0000 | -1.4237 |  | min |
|  |  |  |  |  |  |
|  |  |  | 29 |  | number -ve |
|  |  |  | 39 |  | number +ve |
|  |  |  | 1 |  | number =0 |

### Table 1.4i

In this table the data are as in table 1.4g except that the segregation ratios for plant characters in the F2:F3 experiment and the anthocyanin pigmentation in the trifactorial cross are considered to have a 1:2 segregation ratio.

| **Edwards' Data set assuming 2:1 segregation of plant** | | | | | |
| --- | --- | --- | --- | --- | --- |
| **characters in the F3 of the tri-factorial experiment** | | | | | |
|  |  | **χ^2^** | **χ** |  |  |
|  |  | 84 | 84 |  | Count |
|  |  | 44.7677 | 12.0355 |  | Total |
|  |  | **0.5329** | **0.1433** |  | **Mean** |
|  |  | **0.4334** | **0.5186** |  | **Variance** |
|  |  | 0.6583 | 0.7201 |  | Std Dev |
|  |  | 3.5394 | 1.8813 |  | max |
|  |  | 0.0000 | -1.4237 |  | min |
|  |  |  |  |  |  |
|  |  |  | 33 |  | number -ve |
|  |  |  | 48 |  | number +ve |
|  |  |  | 3 |  | number =0 |
