# Supplementary material for: Mendel’s pea crosses: varieties, traits and statistics
Source: Hereditas. 2019 Oct 31;156:33. doi: 10.1186/s41065-019-0111-y (PMC6823958; doi:10.1186/s41065-019-0111-y)
Supplement: Supplementary file 2 — Additional file 2: The F2:F3 progeny tests for plant characters. Analysis of segregation ratios in Mendel’s F2:F3 progeny tests. [file 41065_2019_111_MOESM2_ESM.docx]

## The F2:F3 progeny tests for plant characters

In the first set of F2:F3 progeny tests of mature plant characters, 10 F3 plants were examined for each F2 individual (Additional file 1, Supplementary Table 1.2). Fisher (1936) was concerned that with just 10 F3 plants there should be a significant amount of misclassification. Fisher's reasoning appears to be as follows:

If among N plants

the number of homozygotes is x

and the number of heterozygotes is y

and we expect y = 2x

so: x = N/3 , y = 2N/3

When 10 F3 plants are examined, then the

expected number classified as heterozygotes is (1 - (0.75^10^)) (2N/3) = 0.6291N

so the expected number classified as homozygotes is (N/3) + (0.75^10^)(2N/3) = 0.3709N

or, instead of 1 : 2 the ratio should be 1.1126 : 1.8874 (≈0.37 : 0.63, ≈1 : 1.6964 )

It should be noted that the term (0.75^x^) is never zero, so the 1:2 ratio should never in fact be achieved, although for values of x > 50 the difference is less than 5x10^-5^% and this error can be reasonably ignored. Additionally, in any segregation experiment, including Mendel’s actual experiments (as Fisher points out), it is unlikely that y = 2x exactly in any given group of F2 plants. Taking the number classified as homozygotes or heterozygotes from Mendel’s paper (Mendel, 1866) we can correct for misclassification using Fisher’s relationship (Supplementary Table 2.1).

### Supplementary Table 2.1. Observed and predicted numbers of homozygotes and heterozygotes

| Number of plants | | | | | | | |  |  |
| --- | --- | --- | --- | --- | --- | --- | --- | --- | --- |
| Mendel's observations | |  | misclassified | |  | Predicted | |  | Character |
| *Aa* | *AA* |  | value | rounded |  | *Aa* | *AA* |  |  |
| 64 | 36 |  | 3.82 | 4 |  | 68 | 32 |  | *A : a* |
| 71 | 29 |  | 4.24 | 4 |  | 75 | 25 |  | *V : v* or *P : p* |
| 60 | 40 |  | 3.58 | 4 |  | 64 | 36 |  | *Gp : gp* |
| 67 | 33 |  | 4.00 | 4 |  | 71 | 29 |  | *Fa : fa* or *Fas : fas* |
| 72 | 28 |  | 4.30 | 4 |  | 76 | 24 |  | *Le : le* |
| 65 | 35 |  | 3.88 | 4 |  | 69 | 31 |  | *Gp : gp* |
|  |  |  | total | 24 |  |  |  |  |  |

The observed numbers are taken from the BSHS translation (Mendel 2016). If the actual number of heterozygotes is *T*, and the chance that an F3 family of 10 individuals derived from a heterozygous F2 fails to segregate any homozygous recessives is *m*, then from Fisher's line of reasoning we have: *m* = (1 - 0.25)^10^. The expected number of misclassified dominant homozygotes is *Tm.* The number of F3 plants Mendel classified as heterozygotes (*N_Aa_*, first column, Mendel's observations under *AA*) where *N_Aa_* = *T*(1 - *m*), so *Tm* = *N_Aa_* (*m*/(1 - *m*)). The value of *Tm* is shown in the column 'misclassified' as its value rounded to the nearest integer. The third column shows the adjusted predictions of heterozygotes and homozygotes, given this number of misclassifications. The character gene designations in the last column are from Ellis et al. (2011).

The possibility of their having been an excess of 24 homozygotes (or deficiency of 24 heterozygotes) corresponds to 24 too few plants with the recessive phenotype among Mendel's 6,000 F3 plants, in this experiment, was the basis of Fisher’s critique (see Table III of Fisher 1936). This lack of homozygous recessives is expected to have led to the misclassification of 24 of the 600 F2s. This is what has caused consternation for the last 80 years and has been the source of a persistent assertion that Mendel set out with the deliberate intention of misleading.

Fisher's interpretation requires that Mendel grew exactly 10 F3 progeny plants from seed collected on exactly 600 F2 plants. This would have required 100% success in germination and survival of Mendel's F3 population, but we know from Mendel's data that between 2 and 7% of his plants failed to germinate, or for some other reason failed to reach maturity (see Additional file 1).

Fisher's proposed discrepancy between Mendel's reported data and the expectation based on his analysis is much smaller that the difference between the reported 6,000 plants and the number among these that should be missing because of a failure to germinate or some other event that prevented the plant from reaching maturity.

If Fisher's interpretation is correct then we should be able to test how the predicted number of plants of the *AA* and *Aa* genotypes compares to the expected 1 : 2 ratio. These χ^2^ and χ values for Mendel's F2:F3 progeny test of plant characters assuming the predicted values in Supplementary Table 2.1 are presented in Supplementary Table 2.2.

### Supplementary Table 2.2. χ^2^ and χ values for the segregation of plant characters in the F3

| Character |  | Fisher's predicted number of | |  |  | | χ^2^ | |  | |  | | χ | |  | |
| --- | --- | --- | --- | --- | --- | --- | --- | --- | --- | --- | --- | --- | --- | --- | --- | --- |
|  |  | hets | homs |  |  | |  | |  | |  | |  | |  | |
| *A : a* |  | 68 | 32 |  |  | | 0.0800 | |  | |  | | 0.2828 | |  | |
| *V : v* or *P : p* |  | 75 | 25 |  |  | | 3.1250 | |  | |  | | 1.7678 | |  | |
| *Gp : gp* |  | 64 | 36 |  |  | | 0.3200 | |  | |  | | -0.5657 | |  | |
| *Fa : fa* or *Fas : fas* |  | 71 | 29 |  |  | | 0.8450 | |  | |  | | 0.9192 | |  | |
| *Le : le* |  | 76 | 24 |  |  | | 3.9200 | |  | |  | | 1.9799 | |  | |
| *Gp : gp* |  | 69 | 31 |  |  | | 0.2450 | |  | |  | | 0.4950 | |  | |
|  |  |  |  |  |  | |  | | expected | |  | |  | | expected | |
|  |  | expected | |  | sum | | 8.3400 | | 6 | |  | | 2.9698 | | 0 | |
|  |  | hets | homs |  | average | | 1.4225 | | 1 | |  | | 0.8132 | | 0 | |
|  |  | ⅔(100) | ⅓(100) |  | | variance | | 2.7748 | | 2 | |  | | 0.9135 | | 1 |
|  |  |  |  |  | std dev | | 1.6658 | | √2 | |  | | 0.9558 | | 1 | |

In this table the predicted numbers of heterozygotes (hets) and homozygotes (homs) in Mendel's test of the segregation of plant characters are shown. This prediction is on the assumption that Mendel observed the plant phenotypes in exactly 10 F3 plants derived from exactly 100 F2 individuals and follows Fisher's estimation of the consequent misclassification rate. The predicted values are taken from Table 2.1 and subject to χ and χ^2^ tests to see if there is any deviation from the 1 : 2 segregation expected in the F2. For each of the six experiments there is 1 degree of freedom (hence the expected average of the χ^2^ values is 1). In this table, the sign of χ is determined according to Edwards (1968a, 2008), where χ is positive if the heterozygous class is in excess.

Supplementary Table 2.2 shows that the segregation ratios we can estimate for these experiments exhibit more variation than we would expect, because the mean χ^2^ is greater than 1. For five of the six F3 families there was an excess of the heterozygous class (Supplementary Tables 2.1 and 2.1), reflected in the χ values that are greater than 1, but the difference from expectation is not significant. (There are six χ values. With 100 F2s it is not possible that χ = 0, so χ must be either positive or negative. There are 64 possible arrangements of 6 positive or negative values, of these 6 have exactly 1 negative value and 7 have either 1 or 0 positive values, so the chance of this result is 6(or 7)/64 or about 10%). In this analysis the values for the χ and χ^2^ tests on the F2 that were heterozygous or homozygous is not in any way exceptional. For both tests their average value is within 1 standard deviation of the expected value and the variances are similar to the expected values, for the χ^2^ the variance is slightly larger than expected, suggesting there is no lack of variation. For the χ value the variance is very close to expectation.

### Allele frequencies in the F2:F3 progeny tests for plant characters

We have F2 data from Mendel's experiments on the segregation of plant characters in the dominant class, so can estimate the ratio of genotypes in these particular seed batches. We know the proportion of the F2 that was *aa* and from this, assuming random mating of the gametes, we can estimate the frequency of the *a* and *A* alleles in this F2 sample because we expect the genotypes *aa*, *Aa* and *AA* to be in the ratio *p*^2^ : 2*pq* : *q*^2^. From the F2 we have a direct estimate of *q*^2^ and from the numbers estimated in Table 1.1 we have the ratio *p*^2^ : 2*pq.* We can therefore ask whether Mendel's values of *p* are the same for the F2 and the F3 data as shown in Supplementary Table 2.3 and Supplementary Figure 2.1. The most extreme deviation from expectation is at about +2 SD units and a deviation of that magnitude is expected in about 2% of single experiments or ca. 12% of sets of six trials as in the present case.

### Supplementary Table 2.3. Allele frequency estimates from the F2 population and F3 families

|  |  | Number of plants | | | |  |  |  |
| --- | --- | --- | --- | --- | --- | --- | --- | --- |
| F2 ratios |  | *XX & Xx* | | *xx* | total |  |  |  |
|  |  | *N* (1 - *p*^2^) | | *N p*^2^ | *N* |  |  | *p* |
| *A : a* |  | 705 | | 224 | 929 |  |  | 0.491 |
| *V : v* or *P : p* |  | 882 | | 299 | 1181 |  |  | 0.503 |
| *Gp : gp* |  | 428 | | 152 | 580 |  |  | 0.512 |
| *Fa : fa* or *Fas : fas* |  | 651 | | 207 | 858 |  |  | 0.491 |
| *Le : le* |  | 787 | | 277 | 1064 |  |  | 0.510 |
|  |  |  |  |  |  |  |  |  |
| from F3 families |  | *XX* | *Xx* |  |  |  |  |  |
|  |  | *N* (1 - *p*)^2^ | 2*N p*(1 - *p*) |  |  | 2*p*/(1-*p*) |  | *p* |
| *A : a* |  | 32 | 68 |  | 100 | 2.125 |  | 0.515 |
| *V : v* or *P : p* |  | 25 | 75 |  | 100 | 3.000 |  | 0.600 |
| *Gp : gp* |  | 36 | 64 |  | 100 | 1.778 |  | 0.471 |
| *Fa : fa* or *Fas : fas* |  | 29 | 71 |  | 100 | 2.448 |  | 0.550 |
| *Le : le* |  | 24 | 76 |  | 100 | 3.167 |  | 0.613 |
| *Gp : gp* |  | 31 | 69 |  | 100 | 2.226 |  | 0.527 |

The relationship between the F2 and F3 estimates of allele frequencies are presented in Supplementary Figure 2.1. Two things are noticeable from this Figure and Table 2.3. The first is that the characters where there is an excess or deficiency of the recessive allele in the F2 do not have a corresponding deficiency or excess of the recessive allele in the sample of 100 used for the generation of the F3 families. However, the deviation is well within the expected range for all the characters except for *le* and *v* (or *p*) and for these two a deviation of that magnitude is expected in at least 10% of data sets such as this. It is noticeable that the deviation for *le* and *v* (or *p*) is of a similar magnitude, which would be consistent with the set of 100 F2 plants from which seed was collected having a similar small excess frequency of heterozygotes, which would necessarily be the case if there was only one F2 population for these two characters.

In his second letter to Nägeli, Mendel discussed a four factor cross where the parents were *ii, aa, VV (&PP), lele* and *II, AA, vv (or pp), LeLe*, commenting that, in 1859 he obtained a descendant with large tasty seeds which bred true and was cultivated in the monastery vegetable garden. Eichling (1942) reported Mendel having said he obtained this tall "shelling type" (*LeLe, PP* & *VV*) from a cross between a "tall sugar-pod type" (*LeLe*, *vv* or *pp*) and a "bush", "shelling type" (*lele, VV* & *PP*). These two comments appear to refer to the same cross, and it is possible that the data from this cross contributed to Mendel's description of the segregation of *le* and *v* (or *p*) in the F2:F3 analysis. We know that Mendel took at least some of the progeny of this cross through several generations, so this material and data was available to him. The expected frequencies of the F2 genotypic classes, assuming a recombination fraction of 0.017 as described by Lamprecht and Mrkos (1950) are shown in the table of expected percentages of different genotypic classes in the F2 of a cross segregating for *Le/le* and *V/v* shown below.

|  | *LeLe* | *Lele* | *lele* |
| --- | --- | --- | --- |
| *VV* | *LeLe VV* | *Lele VV* | *lele VV* |
|  | *0.286* | *4.778* | *19.936* |
| *Vv* | *LeLe Vv* | *Lele Vv* | *lele Vv* |
|  | *4.778* | *40.445* | *4.778* |
| *vv* | *LeLe vv* | *Lele vv* | *lele vv* |
|  | *19.936* | *4.778* | *0.286* |

The *LeLeVV* and *LeleVv* genotypes constitute 40.731%. These individuals are either homozygous dominant for both characters or heterozygous for both characters, so in about 40% of this F2 population the dominant individuals for *Le* are actually the same individuals that are dominant for *V*. In such an experiment *Le* and *V* are not independent.

A second issue is whether such linkage may have been readily apparent to Mendel, so as to cause some concern for his theory. If *le* and *v* were the genes involved and common F2 plants were involved in this analysis then the linkage between *le* and *v* is important. The recombination fraction between *le* and *v*, from the data presented by Lamprecht and Mrkos 1950, calculated according to Allard's equation 6 (Allard 1956), is 0.1067 < *r* < 0.1068; so the expected number of double recessives among 100 F2 progeny is 0.285, as opposed to ca. 6 if the determinants were *le* and *p* i.e. unlinked. Thus we would expect Mendel to have seen 1 or zero double recessives in the F2. At the 1% level, seeing 1 double recessive among 100 F2s when expecting 6.25 is not statistically significant (χ^2^= 4.70, p = 0.0301) but seeing none among 100 is significant (χ^2^= 6.67, p = 0.0098).

### Supplementary Figure 2.1 Recessive allele frequency (*p*) estimated from the F2 and F3 families


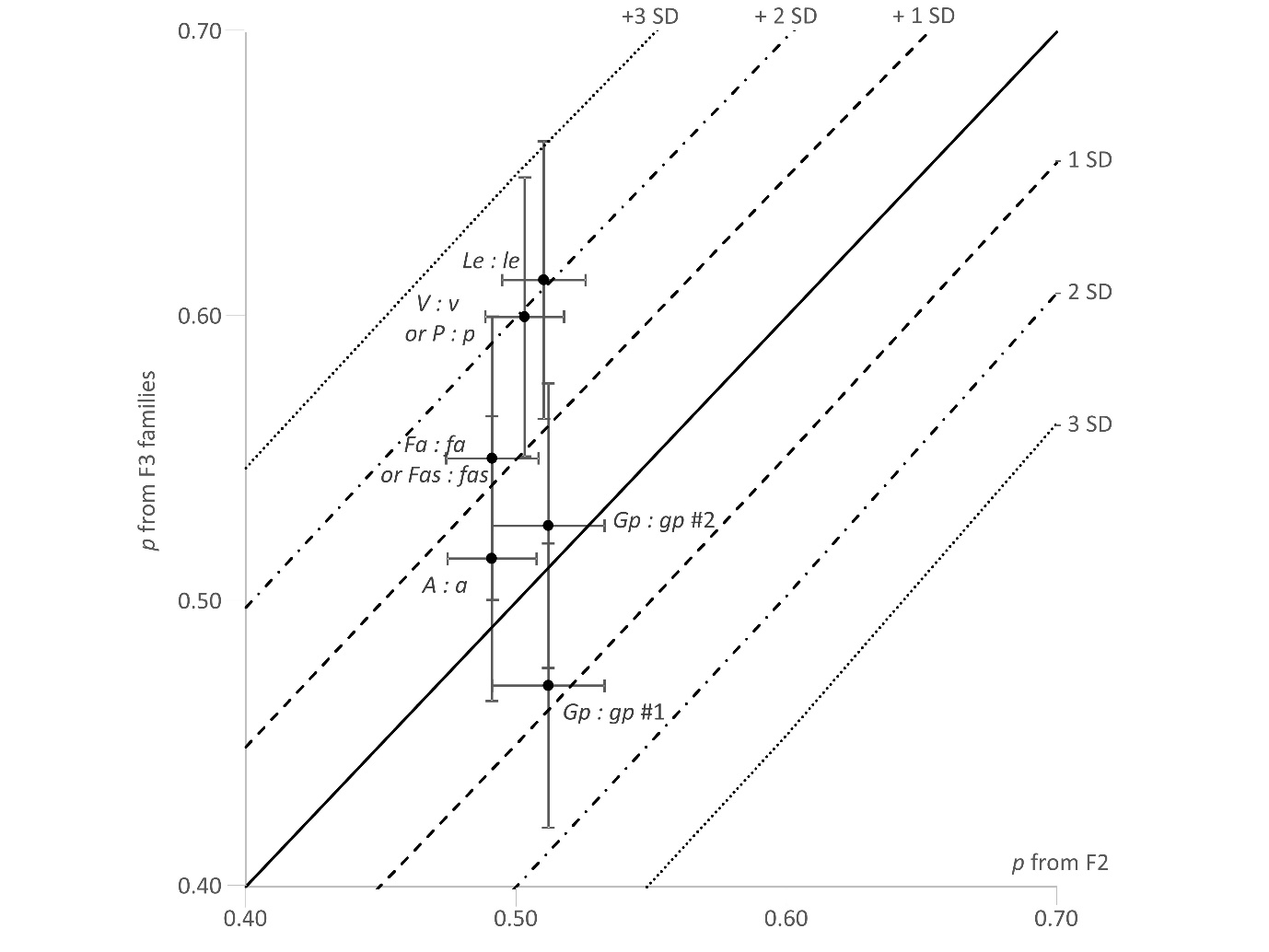


The frequency of the recessive allele in the F2 population of Mendel's experiments on plant characters, as estimated in Supplementary Table 2.3 from the F2 segregation (x axis) is plotted against the corresponding estimate from the segregation in the F3 families derived by selfing the F2 plants with the dominant character. Error bars are √*p*(1 - *p*)/*N.* The solid diagonal line is where *y* = *x*, the broken diagonal lines correspond to ± 1, 2 or 3 SD units where *N* = 100, so points are expected within dashed and dotted line (2 x SD) in about 90% of single trials.

### The segregation of anthocyanin pigmentation in the F3 of Mendel's trifactorial experiment.

Table 1 in the main text presents likelihoods for the segregation ratios for *AA* vs *Aa* in the F2 of Mendel's trifactorial cross. Here the equivalent table for the χ and χ^2^ values are presented.

### Supplementary Table 2.4. Anthocyanin pigmentation segregation in Mendel's trifactorial experiment (χ values).

|  |  | **Observed number** | | |  | **χ** |  |  |  | |  |
| --- | --- | --- | --- | --- | --- | --- | --- | --- | --- | --- | --- |
|  |  |  |  | |  |  |  |  | **Given the F2 ratio** | | |
|  |  |  | | |  |  |  |  | **Misclassification type** | | |
| **genotypic class** |  | ***Aa*** | | ***AA*** |  | **1 : 2** | **Fisher** |  | **None** | **Fisher** | |
| *Rr Ii* |  | 78 | | 49 |  | 1.2549 | 0.3488 |  | 1.4691 | 0.5397 | |
| *Rr II* |  | 38 | | 14 |  | -0.9806 | -1.5174 |  | -0.8554 | -1.4034 | |
| *RR Ii* |  | 45 | | 15 |  | -1.3693 | -1.9383 |  | -1.2369 | -1.8173 | |
| *RR II* |  | 22 | | 8 |  | -0.7746 | -1.1816 |  | -0.6797 | -1.0951 | |
| *Rr ii* |  | 40 | | 20 |  | 0.0000 | -0.6020 |  | 0.1415 | -0.4747 | |
| *RR ii* |  | 17 | | 9 |  | 0.1387 | -0.2610 |  | 0.2328 | -0.1766 | |
| *rr Ii* |  | 36 | | 19 |  | 0.1907 | -0.3903 |  | 0.3275 | -0.2676 | |
| *rr II* |  | 25 | | 8 |  | -1.1078 | -1.5276 |  | -1.0102 | -1.4382 | |
| *rr ii* |  | 20 | | 10 |  | 0.0000 | -0.4257 |  | 0.1001 | -0.3357 | |
|  |  |  | |  |  |  |  |  |  |  | |
| *Total* |  | 321 | | 152 |  | -0.5527 | -2.2297 |  | -0.1591 | -1.8749 | |
|  |  |  | |  |  |  |  |  |  |  | |
| *Mean* |  |  | |  |  | -0.2942 | -0.8328 |  | -0.1679 | -0.7188 | |
| *Variance* |  |  | |  |  | 0.6894 | 0.5541 |  | 0.7300 | 0.5778 | |

The number of heterozygotes (*Aa*) and homozygotes (*AA*) observed in Mendel's F2:F3 progeny test for anthocyanin pigmentation in the F3 of Mendel's trifactorial cross is presented for each of the nine genotypic classes according to the seed characters (as in Edwards 1986a, 2008). The χ value for this calculated based on four possible interpretations of the data as discussed in the main text.

### Supplementary Table 2.5. Anthocyanin pigmentation segregation in Mendel's trifactorial experiment (χ^2^ values)

|  |  | **Observed number** | | |  | **χ^2^** |  |  |  | |  |
| --- | --- | --- | --- | --- | --- | --- | --- | --- | --- | --- | --- |
|  |  |  |  | |  |  |  |  | **Given the F2 ratio** | | |
|  |  |  | | |  |  |  |  | **Misclassification type** | | |
| **genotypic class** |  | ***Aa*** | | ***AA*** |  | **1 : 2** | **Fisher** |  | **None** | **Fisher** | |
| *Rr Ii* |  | 78 | | 49 |  | 1.574800 | 0.121700 |  | 2.1584 | 0.291200 | |
| *Rr II* |  | 38 | | 14 |  | 0.961500 | 2.302600 |  | 0.7316 | 1.969400 | |
| *RR Ii* |  | 45 | | 15 |  | 1.875000 | 3.757200 |  | 1.5299 | 3.302500 | |
| *RR II* |  | 22 | | 8 |  | 0.600000 | 1.396300 |  | 0.4620 | 1.199300 | |
| *Rr ii* |  | 40 | | 20 |  | 0.000000 | 0.362400 |  | 0.0200 | 0.225400 | |
| *RR ii* |  | 17 | | 9 |  | 0.019200 | 0.068100 |  | 0.0542 | 0.031200 | |
| *rr Ii* |  | 36 | | 19 |  | 0.036400 | 0.152300 |  | 0.1072 | 0.071600 | |
| *rr II* |  | 25 | | 8 |  | 1.227300 | 2.333600 |  | 1.0206 | 2.068500 | |
| *rr ii* |  | 20 | | 10 |  | 0.000000 | 0.181200 |  | 0.0100 | 0.112700 | |
|  |  |  | |  |  |  |  |  |  |  | |
| *Total* |  | 321 | | 152 |  | 0.305500 | 4.971700 |  | 0.0253 | 3.515100 | |
|  |  |  | |  |  |  |  |  |  |  | |
| *Mean* |  |  | |  |  | 0.6994 | 1.1862 |  | 0.6771 | 1.0302 | |
| *Variance* |  |  | |  |  | 0.5483 | 1.7947 |  | 0.5848 | 1.3881 | |

The number of heterozygotes (*Aa*) and homozygotes (*AA*) observed in Mendel's F2:F3 progeny test for anthocyanin pigmentation in the F3 of Mendel's trifactorial cross is presented for each of the nine genotypic classes according to the seed characters (as in Edwards 1986a, 2008). The χ^2^ value for this calculated based on four possible interpretations of the data as discussed in the main text.

## Additional file 2 Reference

Allard RW (1956) Formulas and tables to facilitate the calculation of recombination values in heredity. Hilgardia 24:235-277
