# Supplementary material for: Mendel’s pea crosses: varieties, traits and statistics
Source: Hereditas. 2019 Oct 31;156:33. doi: 10.1186/s41065-019-0111-y (PMC6823958; doi:10.1186/s41065-019-0111-y)
Supplement: Supplementary file 3 — Additional file 3: Mendel’s F2:F3 progeny test experiments. Discussion of how Mendel’s F2:F3 progeny test experiments may have been performed given the material that would have been available. [file 41065_2019_111_MOESM3_ESM.docx]

### Mendel's F2:F3 progeny test experiments

Here we discuss how Mendel might have conducted the experiment which he described as:

"For each of the following trials, 100 plants were selected that possessed the dominating trait in the first generation, and in order to test its meaning, 10 seeds were cultivated from each of the selected plants." p16

and as illustrated in Figure 2 of the main text. Seidenfeld (2008) proposed a scheme, following the practice of "any experienced gardener" by planting more seeds and then thinning the plants to the required number. Here we examine Mendel’s data to estimate plant survival rate and therefore the probability of his obtaining six experiments with 100 F3 families where exactly 10 seeds were sown and all survived. We suggest an experimental strategy that Mendel might have used.

In section 8 of the 1866 paper Mendel wrote that in his bifactorial cross he obtained 556 seeds, of which 11 did not germinate, and of those that did, 3 failed to set seed. In the trifactorial experiment, 639 plants, from 687 seeds, reached maturity. This allows us to estimate that Mendel had losses of somewhere between 2 and 7% from his seed lots. This would suggest that if he selected exactly 10 seeds from exactly 100 F2 plants he would, because of this failure rate, be unlikely to obtain 10 segregants in all 100 of his F3 families, yet in this experiment all six sets of results had exactly 100 F2 individuals genotyped.

If the probability that a seed does not germinate, or produce a plant that can exhibit the relevant mature phenotype, is taken to be 0.02, this means that the chance that all of 10 seeds survive to reach the required degree of maturity is 0.98^10^, and the chance that not all 10 seeds reach this level of maturity is 1-(0.98^10^) = *s*. The chance that all 10 of the seeds do survive is (1-*s*) and the chance that this happens for 100 sets of 10 such seeds is therefore (1 - *s*)^100^ which is very unlikely (ca. 10^-9^).

How might this experiment have been done in the face of such an unlikely probability and in such a way that did not contradict Mendel's statement? If Mendel sowed more than 10 F3 seeds, say a dozen, from at least a few more than 100 F2 individuals (e.g. 120) then he would be reasonably confident to have 100 F3 families with 10 seeds in each. He could then classify 100 of these F3 families. In addition, he could reasonably adopt the rule that *any family with no recessive segregants and at least 10 plants of the dominant class* would be scored as having been derived from the A (=*AA*) class. For example if there were 9 F3 plants and one was *aa*, the genotype of a 10^th^ would be irrelevant for the purpose of classification, but if there were 9 F3 plants all with the dominant phenotype that family would not be classified. In this scheme, some families may have more than 10 plants with only one recessive. Mendel could not classify these as homozygous dominant and would either classify them correctly or ignore the family and examine another.

If this was in fact the way that Mendel conducted the experiment then the statistical tests that Fisher (and Edwards) performed are overly restrictive.

#### Seed yield in the F2:F3 tests involving R vs r and I vs i

In Mendel's first two experiments, the single factor segregations of *R* vs *r* and *I* vs *i* Mendel obtained 7324 F2 seed from 253 F1 plants in the first experiment and 8023 F2 seeds from 258 F1 plants in the second experiment, or about 30 seeds per plant. Note that the 24 single plant values he gave for these two experiments have an average of 44.21 ± 21.74, μ ± SD which is slightly higher than the average.

If we take 30 seeds per plant as an estimate of Mendel's typical seed yields then in the F2:F3 experiment involving round vs wrinkled seeds and yellow vs green cotyledons then we van estimate the total number of seeds classified in the F2:F3 experiment that involved 565 F2 individuals that were *RR* or *Rr* and 519 F2 individuals that were *II* or *Ii.* That gives 1084 x 30 = 32520, so we can be reasonably confident that this experiment involved the classification of ca. 30,000 seeds.

We can also estimate roughly how many of the F2 individuals had fewer than 10 seeds per plant.

With a mean and standard deviation of 44.21 and 21.74 seeds per plant respectively then approximately 63 plants (5.8%) would have fewer than 10 seeds per plant. If we assume that the coefficient of variation (SD/μ) in the estimate of the mean for the 24 single plants is the same as the coefficient of variation for the experiment as a whole then we can estimate the standard deviation in these experiments as a whole as 14.75. For a normal distribution with a mean of 30 and standard deviation of 14.75 approximately 8.76% of plants (or ca. 95 out of 1084) would have 10 or fewer seeds.
