# Supplementary material for: Mendel’s pea crosses: varieties, traits and statistics
Source: Hereditas. 2019 Oct 31;156:33. doi: 10.1186/s41065-019-0111-y (PMC6823958; doi:10.1186/s41065-019-0111-y)
Supplement: Supplementary file 4 — Additional file 4: Normal probability plots. Analysis of the extent of deviation of segregation ratios in Mendel’s data [11] from a standard normal distribution. [file 41065_2019_111_MOESM4_ESM.docx]

## Normal probability plots

Edwards (1986a, 2008) tabulated χ values for Mendel's segregation data (Additional file 1, Table 1.3) and plotted these χ values in a normal probability plot (i.e. where the x axis is the cumulative probability of χ) and used this to illustrate a deficiency of extreme segregants in Mendel's data. It should be noted that in this plot the lowest and highest χ values are missing because they correspond to the cumulative probabilities of 0 and 1. The plot is also influenced by the sign of χ, which Edwards assigned on the basis of two rules: (i) an excess of the class with more expected segregants is assigned a +ve value of χ, (ii) where the segregation ratio is expected to be 1 : 1 the class with more dominant alleles is assigned a +ve value of χ. Only the second of these rules is required and if it is applied consistently some of the χ values change sign.

The problem of how to assign +ve and -ve values can be overcome by plotting the absolute value of χ, and this also adds an additional point to the plot. A further difference from Edwards' plot is encountered if Mendel's four extreme values are included.

These considerations beg the question of which segregation ratios should be included in an analysis. If the 'extreme' values are excluded because they are a non-random sample, then the same should apply to the first ten plants from these two experiments: they are necessarily a non-random sample and we know from the data Mendel presented that they had a higher seed yield than was generally the case in these two experiments. This is consistent with their being 'unconsciously selected as the best plants' as Rasmusson suggested to Fisher (1936). Thus we can either take Mendel's data as a whole, or, we can consider only the consolidated values for the first two experiments. If we adopt the latter approach then there are 64 ratios to be considered (Additional file 1, Table 1.3). They have an average χ^2^ of 0.57 ± 0.83 (mean ± SD), as compared to an expected 1 ± 1.414, and the mean is within one standard deviation of expectation. We can also ask what proportion of values are above or below the expected median (ca. 0.4705). The number of ratios observed above the median is 42, and below is 22 (vs the expected 32 for each); the significance for a chi-squared test of these observed vs expected ratios is not significant at *p* = 0.0124, suggesting that this distribution is unremarkable; that the low mean value is easily understood as a consequence of the non-symmetrical distribution of expected χ^2^ values.

We can further ask whether these 64 values follow the expected standard normal distribution of χ, and this is described below for |χ|, where a small but significant deviation from the standard normal distribution is observed.

### Normal probability plots

The cumulative distribution of χ values is illustrated in Supplementary Figure 4.1, for this purpose χ values have to be given a sign according to some convention. In the figure, the graph on the left corresponds to the assignment of sign of χ as used by Edwards, while the plot on the right uses the single consistent rule where an excess of the genotypic class with the larger number of dominant alleles is assigned a positive value. Applying that single rule changes the distribution of χ values as is shown by comparing the two plots. The way this sign is determined can have a consequence for the interpretation of the data, so we might attribute something to Mendel's data that is really a consequence of the way we choose the sign of χ.


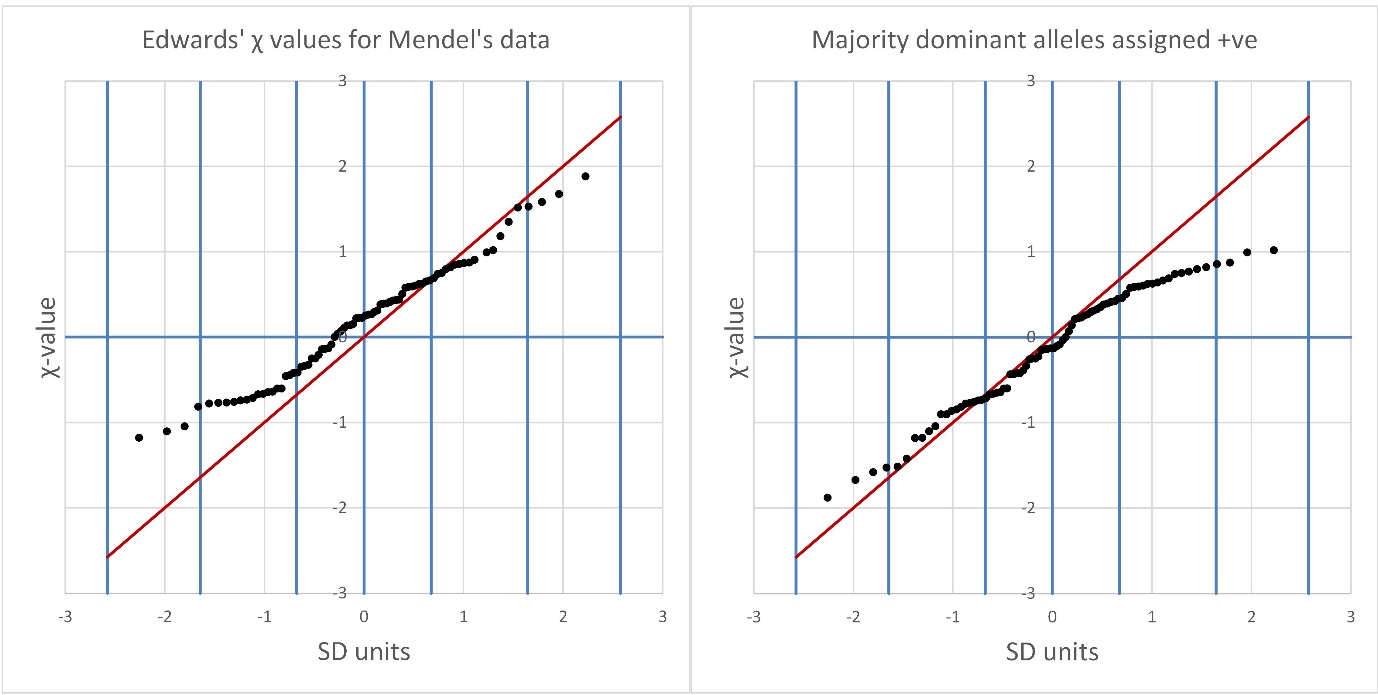


### Supplementary Figure 4.1 assigning the sign of χ

The figures plot the values of χ (y axis) in ascending order. Their position on the x axis corresponds to the cumulative probability. If there are *n*+1 values then there are *n* increments such that the probability increments by 1/*n* for each successive value. The cumulative probability is converted to standard deviation units with the Excel function NORM.INV, and these values are used on the x axis. The vertical blue lines correspond to the cumulative probabilities 0.5, 0.75, 0.95 and 0.995. The red diagonal line corresponds to the standard normal distribution between the outermost vertical blue lines. The figure on the left corresponds to the assignment of sign of χ as used by Edwards, while the plot on the right uses the single consistent rule where the genotypic class with the larger number of dominant alleles is assigned a positive value.

The difference between these two plots suggests that the rule for assigning χ may affect the interpretation, but this problem can be overcome by considering the absolute value of χ. These values are all in the positive half of the expected standard normal distribution and correspond to the positive half of that distribution, this is shown in Supplementary Figure 4.2.

### Supplementary Figure 4.2 probability plot for |χ|


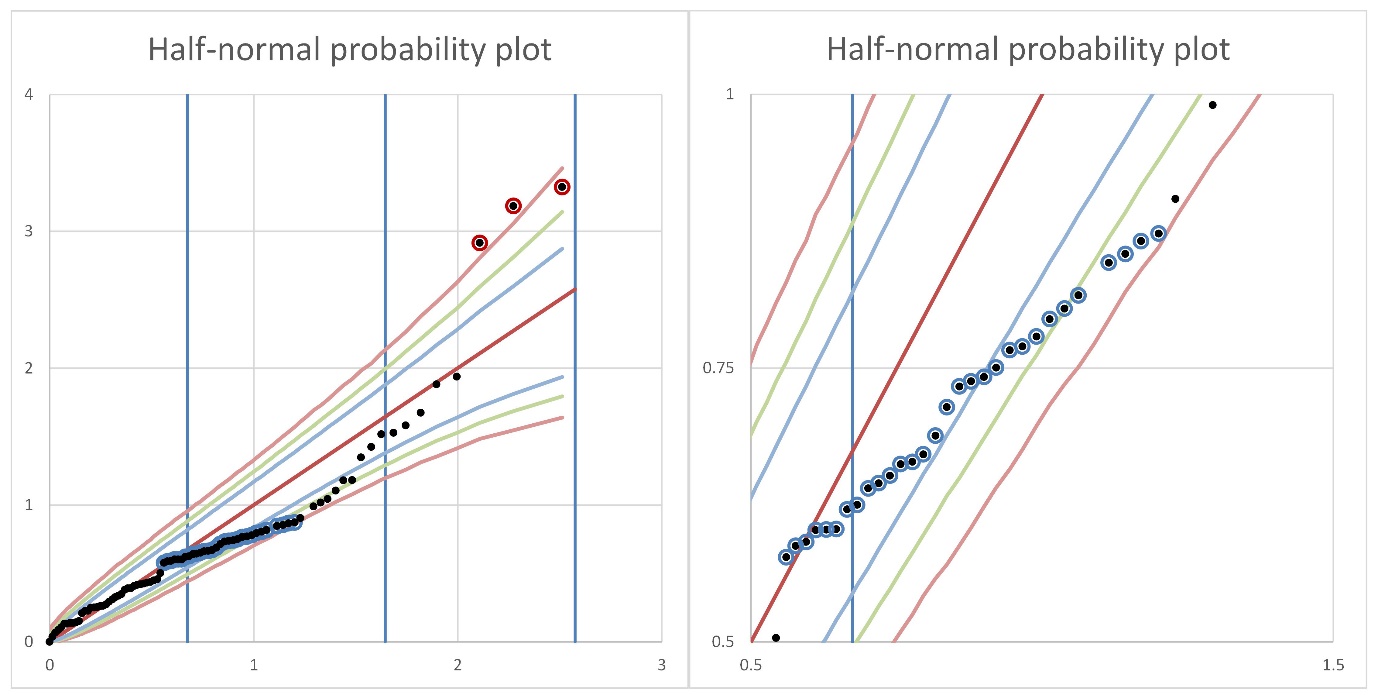


This figure (left) plots the |χ| values for all of Mendel's data (black dots). The four extreme segregation ratios were included in the data set, but the most extreme value does not appear on the plot as it corresponds to the 100^th^ percentile and is therefore at an arbitrarily large value on the x axis. The three extreme values that do appear on the plot are ringed in red. The vertical blue lines correspond to the 0.5, 0.75, 0.95 and 0.995 percentile points. The expected values for the standard normal distribution fall on the diagonal red line. Confidence intervals are plotted for 0.1% (pink), 1% (green) and 5% (light blue). These were estimated from 100,000 sets of 88 standard normal variates generated in R. The figure on the right expands a region of the graph.

There is a run of |χ| values from 0.57 to 0.90 (ringed in blue) that draws the points to the right, eventually to the 0.5% confidence interval at the final point of that sequence. This final point alone is not necessarily responsible for this deviation, rather it is that there are more |χ| values within this range than expected from the standard normal distribution. These correspond to the range 0.33 < χ^2^ < 0.76 which includes the median value of a χ^2^ distribution with one degree of freedom.

Two of the four "extreme" values are outside the 99.5% confidence limit, and between these two places where the cumulative distribution is beyond the most extreme confidence limits the values return to the expected range.

The analysis illustrated in Supplementary Figures 4.1 and 4.2 suggests that Mendel's data do not conform to the expected standard normal distribution of χ values. Perhaps this is not surprising because the data include values Mendel used to illustrate extremes of segregation and the non-random sample of the first 10 plants from experiments 1 and 2. While both these sets of 10 plants and the extreme segregation ratios are illustrative of variation, on balance it seems better to analyse the segregation ratios of the undivided group of seeds for these two experiments. If we restrict analysis to this data set then there are 64 segregation ratios to consider and these data have an average χ^2^ = 0.57 ± 0.83 (μ ± SD, compared to the expected 1 ± 1.14). We can ask how these 64 χ^2^ values compare with expectation, this is shown in Figure 3 of the main text and the corresponding values are given in Supplementary Table 4.1.

### Supplementary Table 4.1 The frequency distribution of χ^2^ values

|  | Mendel's data set | | n = 64 |  | Complete data set | | n = 88 |  | Complete data set | | 84 |
| --- | --- | --- | --- | --- | --- | --- | --- | --- | --- | --- | --- |
| χ^2^ range | Obs | Exp | χ^2^ |  | Obs | Exp | χ^2^ |  | Obs | Exp | χ^2^ |
| 0 - 0.5 | 43 | 33.31 | 2.82 |  | 52 | 45.80 | 0.84 |  | 52 | 43.72 | 1.57 |
| 0.5 - 1 | 12 | 10.38 | 0.25 |  | 19 | 14.27 | 1.57 |  | 19 | 13.62 | 2.12 |
| 1 - 1.5 | 3 | 6.18 | 1.64 |  | 5 | 8.50 | 1.44 |  | 5 | 8.12 | 1.20 |
| 1.5 - 2 | 0 | 4.06 |  |  | 1 | 5.58 | 3.76 |  | 1 | 5.32 | 3.51 |
| 2 - 2.6 | 3 | 2.78 |  |  | 4 | 3.82 |  |  | 4 | 3.65 |  |
| 2.5 - 3 | 1 | 1.96 |  |  | 1 | 2.69 |  |  | 1 | 2.57 |  |
| 3 - 3.5 | 0 | 1.40 |  |  | 0 | 1.93 |  |  | 0 | 1.84 |  |
| 3.5 - 4 | 2 | 1.02 |  |  | 2 | 1.40 |  |  | 2 | 1.33 |  |
| >4 | 0 | 2.91 |  |  | 4 | 4.00 |  |  | 0 | 3.82 |  |
|  |  |  |  |  |  |  |  |  |  |  |  |
| >1.5 | 6 | 14.12 | 4.67 |  | 12 | 19.42 | 2.83 |  | 8 | 18.54 | 5.99 |
| ∑ |  |  | 9.38 |  |  |  | 6.68 |  |  |  | 10.88 |
| *p* |  |  | 0.025 |  |  |  | 0.083 |  |  |  | 0.012 |

Although some classes are in excess and others deficient with respect to expectation the overall χ^2^ values for the deviation of these distributions from expectation is not significant at the 1% level. We can further ask whether these values are or are not distributed as expected for a standard normal distribution, and this is presented as an analysis of the cumulative distribution of the absolute values of χ in in Supplementary Figure 4.2 for the complete data set and Supplementary Figure 4.3 for the 64 values without disaggregation.

### Supplementary Figure 4.3 probability plot for |χ| of 64 segregation ratios in Mendel's data


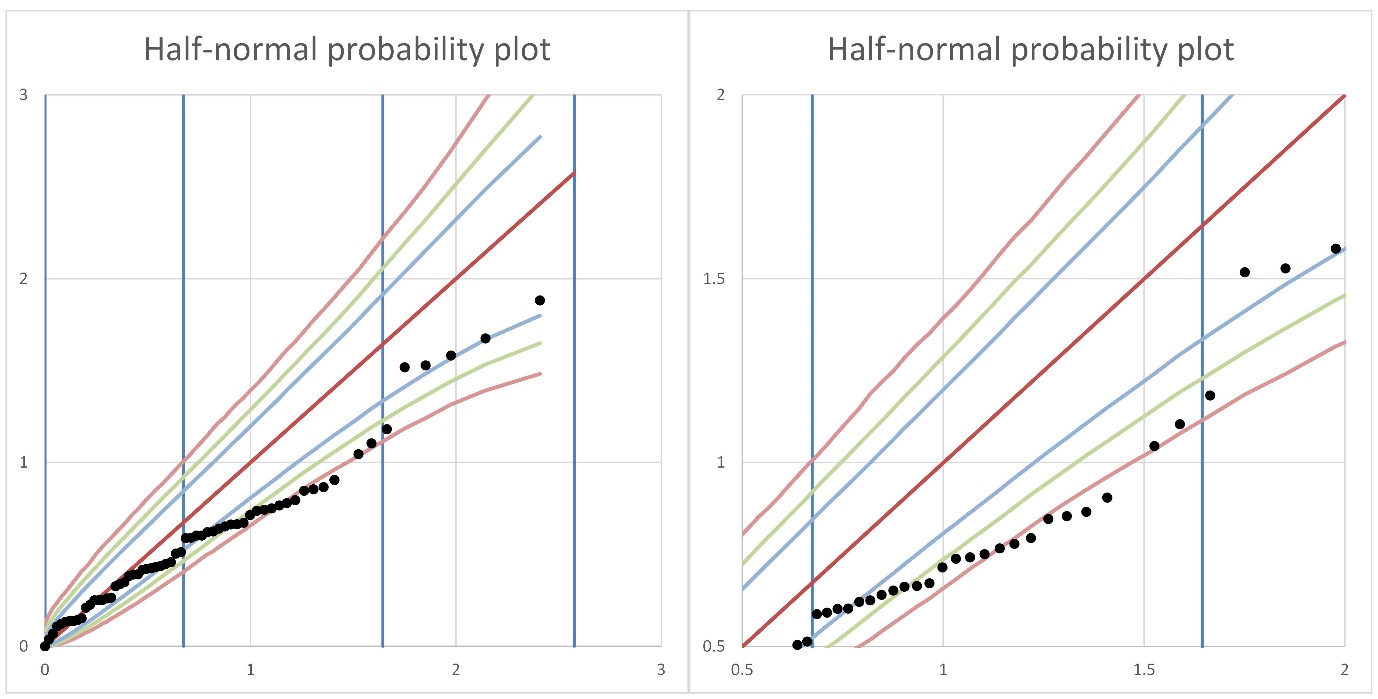


This figure (left) plots the |χ| values for all of Mendel's data (black dots) where the four extreme segregation ratios and twenty individual plant segregations are combined with the total data for experiments 1 and 2. The vertical blue lines correspond to the 0.5, 0.75, 0.95 and 0.995 percentile points. The expected values for the standard normal distribution fall on the diagonal red line. Confidence intervals are plotted for 0.1% (pink), 1% (green) and 5% (light blue). These were estimated from 100,000 sets of 64 standard normal variates generated in R. The figure on the right expands a region of the graph.

In this plot six values are outside the 0.5% confidence interval (two are very close, but just inside the interval). Again this is a result of there being slightly more than expected |χ| values in the range 0.59 to 1.10.

Figures 4.2 and 4.3 show that the choice of the way the data is partitioned or aggregated has a consequence for the distribution of data values.
